# Effects of hypothermic oxygenated machine perfusion on bile composition after liver transplantation – Findings from a randomized controlled trial

**DOI:** 10.1016/j.jhepr.2025.101647

**Published:** 2025-10-17

**Authors:** Frederik Schliephake, Isabella Lurje, Deniz Uluk, Janina Eden, Zoltan Czigany, Justus Pein, Peri Husen, Cornelius Engelmann, Christoph Michalski, Marlene Kohlhepp, Pavel Strnad, Philipp Dutkowski, Frank Tacke, Ulf Peter Neumann, David Meierhofer, Georg Lurje

**Affiliations:** 1 Department of Surgery, Charité – Universitätsmedizin Berlin, Germany; 2 Department of General-, Visceral- and Transplantation Surgery, Heidelberg University Hospital, Heidelberg, Germany; 3 Department of Hepatology and Gastroenterology, Campus Charité Mitte | Campus Virchow-Klinikum, Charité – Universitätsmedizin Berlin, Berlin, Germany; 4 Department of Surgery, Section of HPB Surgery and Liver Transplantation, University of Groningen and University Medical Center Groningen, Groningen, The Netherlands; 5 Department of Surgery and Transplantation, University Hospital RWTH Aachen, Aachen, Germany; 6 Department of Internal Medicine III, University Hospital RWTH Aachen, Aachen, Germany; 7 Department of Surgery, University Hospital Basel, Basel, Switzerland; 8 Max Planck Institute for Molecular Genetics, Mass Spectrometry Facility, Berlin, Germany

**Keywords:** Hypothermic oxygenated machine perfusion (HOPE), Bile transporters, Biliary phospholipids, Bile acid composition, Liver transplantation (LT), Ischemia–reperfusion injury (IRI)

## Abstract

**Background & Aims:**

In liver transplantation, bile acid (BA) toxicity contributes to injury of both hepatocytes and cholangiocytes. While hypothermic oxygenated machine perfusion (HOPE) reduces ischemia–reperfusion injury (IRI) and improves clinical outcomes, its impact on bile composition remains unclear because of the lack of bile samples available after LT.

**Methods:**

Bile, blood, and liver tissue were collected within a multicentric randomized controlled trial (NCT03124641), from 26 patients receiving extended criteria donation (ECD) allografts from donors after brain death (DBD). Fourteen donor livers were static cold stored (SCS group), while 12 livers underwent end-ischemic HOPE. Grafts were randomly assigned. BA levels and metabolic parameters were analyzed across samples with mass spectrometry. Expression of bile transporters and enzymes was assessed in liver biopsies before and after transplantation.

**Results:**

Serum and biliary levels of hydrophobic BAs were positively correlated with IRI severity, such as serum aspartate aminotransferase and alanine aminotransferase, and decreased across postoperative days (POD) for all allografts (bile: POD-1 *vs.* POD-2/-3, *p* <0.001; blood: admission *vs.* POD-1–3, *p* <0.001). Expression of the hepatocyte bile transporters ABCB4 and ABCG8 decreased post-reperfusion (*p* = 0.045; *p* <0.001). In the SCS group, intrahepatic BA levels increased post-reperfusion (8.71 to 10.77 pmol/mg, *p* = 0.023), while biliary BA levels decreased postoperatively (POD-1 *vs.* POD-3, *p* = 0.06). The HOPE group showed higher biliary BA and phosphatidylcholine (PC) levels on POD-3 compared with the SCS group (total BAs: 2.30 × 10^9^*vs.* 2.03 × 10^9^ peak area, *p* = 0.047; PC: 6.49 × 10^8^*vs.* 4.48 × 10^8^ peak area, *p* = 0.031), and a decline in biliary BA/PC ratio.

**Conclusions:**

This is the first randomized study demonstrating effects of HOPE treatment on bile composition following ECD-DBD liver transplantation. Protection from BA toxicity may represent a novel mechanism underlying the effects of HOPE.

**Clinical trials registration:**

This trial is registered at ClinicalTrials.gov (NCT03124641).

**Impact and implications:**

Worldwide liver allograft scarcity has led to the implementation of hypothermic oxygenated machine perfusion (HOPE) to enhance the safety of liver transplantation (LT) using extended criteria donors. However, its protective mechanisms remain incompletely understood, largely because of limited human data. In this study, we provide evidence that HOPE improves biliary lipid secretion after LT, limits intrahepatic bile acid accumulation upon allograft reperfusion, reduces the biliary bile acid to phosphatidylcholine ratio and promotes normalization of serum bile acids levels post-LT. Our findings suggest that protection from bile acid toxicity may constitute a novel mechanism underlying the effects of HOPE.

## Highlights

• Hydrophobic bile acid levels in bile and serum correlate with AST/ALT after liver transplant and decline postoperatively.
• Expression of the hepatocyte bile transporters ABCG8 and ABCB4 decreases after reperfusion.
• In cold-stored organs, intrahepatic bile acids accumulate following reperfusion.
• HOPE-treated livers show faster recovery of biliary bile acid and phospholipid secretion, with earlier normalization of serum bile acid levels.

## Graphical abstract

**Figure:**
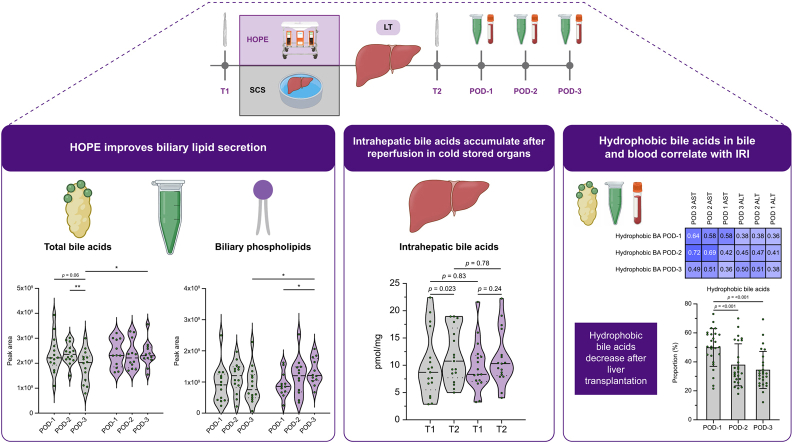

## Introduction

The global demand for liver transplantation (LT) far exceeds allograft availability, leading to a critical shortage of organs. This scarcity has driven a paradigm shift towards the utilization of allografts from extended criteria donors (ECD), which include organs from older donors, those with comorbidities, or those that have sustained prolonged ischemic times.^1^ While the adoption of ECD organs increases the donor pool, it also introduces additional challenges, including higher risks of graft dysfunction and complications, resulting from an increased susceptibility of ECD organs towards ischemia–reperfusion injury (IRI).[2], [3], [4]

Bile production and composition reflect the functional integrity of the hepatobiliary system and can be impaired in ECD allografts.^5^ Bile acids are the major biliary solute component and are synthesized by the hepatocytes as primary bile acids (PBAs), predominantly in the form of cholic acid (CA) and chenodeoxycholic acid (CDCA).^6^^,^^7^ They are secreted into the biliary system through ATP-dependent transporters and recirculate to the liver after intestinal reabsorption. A small portion of PBAs is transformed by gut bacteria into secondary bile acids (SBAs), which either follow their precursors into the enterohepatic circulation or are excreted in feces.^7^ PBAs and SBAs together form a systemic bile acid pool, although their relative distribution varies across tissues and compartments.^6^ In human bile, PBAs account for approximately 80% of the bile acid pool, while deoxycholic acid (DCA) and ursodeoxycholic acid (UDCA) represent the most abundant SBAs forms.^8^ Despite being crucial for bile formation, digestion of dietary lipids and elimination of cholesterol, bile acids may exhibit cytotoxic, profibrotic,^9^ and proinflammatory effects,^10^ contributing to the pathogenesis of a various liver diseases.^11^ Bile acid toxicity results primarily from hydrophobic forms, through disruptions of the cell membrane,^12^ mitochondrial damage,^13^ and promotion of inflammatory responses,^9^ leading to hepatocellular and cholangiocellular injury. Conversely, hydrophilic bile acids such as UDCA and biliary phospholipids counteract these cytotoxic effects.^14^^,^^15^

In the context of LT, ischemia–reperfusion injury, and intracellular ATP depletion during transplantation have been implicated in impairing biliary lipid secretion, leading to intrahepatic accumulation of cytotoxic bile acid species and biliary lack of cytoprotective phosphatidylcholine.^16^ An increased bile acid to phosphatidylcholine ratio has been shown to induce bile duct injury^17^^,^^18^ and is associated with the development of non-anastomotic strictures (NAS),^19^ a severe biliary complication following LT. Interestingly, recent preclinical findings suggest that hepatic IRI induces a compensatory reprogramming of bile acid metabolism, resulting in a more hydrophilic and anti-inflammatory bile acid composition.^20^ However, bile acid homeostasis after LT, including the influence of IRI on bile acid composition and toxicity, remains unclear, particularly in the context of ECD transplantation.

To improve the quality of ECD organs, innovative preservation techniques such as hypothermic oxygenated machine perfusion (HOPE) have emerged.^21^ HOPE perfuses the organ with an oxygenated solution at low temperatures, which helps to mitigate IRI by maintaining cellular metabolism at a reduced rate and by improving mitochondrial function.^22^ Several randomized controlled trials (RCTs) have provided evidence of better post-transplant organ function and decreased post-transplant complications after HOPE.[23], [24], [25] The therapeutic benefits seem to also include decreased biliary complications,^26^ particularly the development of NAS in allografts donated after circulatory death.^27^ While the effect of HOPE on hepatic IRI is increasingly understood – encompassing mitochondrial protection and reduction of downstream hepatocellular injury^16^^,^^28^ – its influence on bile composition, particularly bile acid metabolism and cytotoxicity after human LT has not been thoroughly investigated yet.

Here, we assess the impact of HOPE on bile composition in ECD-donation after brain death (DBD) livers and investigate the relation between hepatic IRI and the postoperative bile acid pool, by analyzing samples acquired during the first multicenter RCT on HOPE in ECD allografts from DBD (HOPE ECD-DBD). This study contributes to an understanding of HOPE treatment effects in ECD liver allografts as well as the bile acid composition after LT.

## Patients and methods

### Trial and patients

This translational study was conducted with samples acquired from the HOPE ECD-DBD multicentric RCT (ClinicalTrials.gov NCT03124641) that compared HOPE *vs.* static cold storage (SCS) between September 2017 and September 2020.^23^ The investigator-initiated, open-label RCT and the associated sampling protocols were approved by the local ethics committee of the leading center (University Hospital RWTH Aachen; EK 049/17). The study protocol,^29^ the primary and secondary study outcomes,^23^ and clinical long-term results^30^ have been published previously. Briefly, DBD allografts were included if they fulfilled at least one of the following ECD criteria, adapted from the German Medical chamber.^31^

1. Donor age ≥65 years.
2. Donor intensive care therapy before donation for ≥7 days.
3. Donor BMI >30 kg/m^2^.
4. Histological proof of liver steatosis: >40% macrosteatosis or mixed steatosis.
5. Serum-Na^+^ >165 mmol/L.
6. Serum transaminases >3 × upper limits of normal.
7. Serum-bilirubin >2 mg/dl.

After stratified randomization, livers were transplanted without additional interventions according to institutional protocol (SCS group, ‘control’), or after additional end-ischemic HOPE preservation (HOPE group, ‘intervention’). Extended characteristics of the HOPE ECD-DBD trial are reported elsewhere.^23^^,^^30^ All patients included in the analysis received a daily UDCA supplementation of 500 mg during the sampling period post-LT, as part of the standardized institutional LT protocols.

### Blood, bile, and tissue sampling

**Fig. 1:**
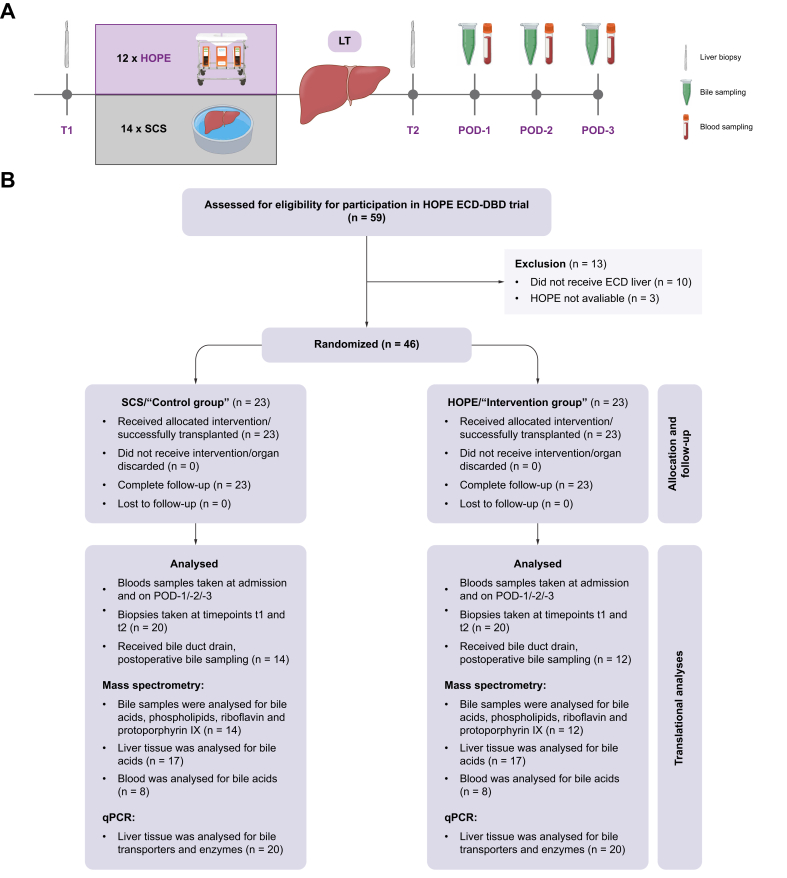
Trial design and translational sampling in the HOPE ECD-DBD trial. (A) Flow chart of perioperative sampling: liver biopsies and bile collection on POD-1–3; blood sampling for routine liver function tests on POD-1–3. (B) Trial flow chart with translational analyses. Modified from Parente *et al.*^28^ ECD, extended criteria donation; HOPE, hypothermic oxygenated machine perfusion, LT, liver transplantation; POD, postoperative day; SCS, static cold storage; T, time point.

Bile samples were acquired on the first 3 days after LT through a routinely, intraoperatively inserted biliary T-drainage. Blood draws were performed on the highlighted days. Liver biopsies were taken during backtable preparation of the allografts (t1) and after organ reperfusion (t2) (Fig. 1A). All samples were snap-frozen with liquid nitrogen and stored at -80 °C. The median storage time for all samples was 67 months (IQR: 54–77 months), with comparable durations between HOPE-treated organs (68 months, IQR: 51–77 months) and the control group (67 months, IQR: 58–75 months).

### Liquid chromatography with tandem mass spectrometry

Bile acid standards including CA, glycoursodeoxycholic acid (GUDCA), lithocholic acid (LCA), glycodeoxycholic acid (GDCA), DCA, taurocholic acid (TCA), tauroursodeoxycholic acid (TUDCA), and taurochenodeoxycholic acid (TCDCA) as well as the deuterium-labeled internal standards (d4-CA, d4-CDCA, and d4-DCA) were obtained from Cayman Chemicals (Ann Arbor, MI, USA). 1,2-Dilauroyl-sn-glycero 3 phosphocholine 12:0 PC (DLPC) and 1,2-diarachidoyl-sn-glycero-3-phosphocholine 20:0 (PC) were purchased from Avanti (Alabaster, AL, USA). Acetonitrile, methanol, and chloroform were from VWR (Darmstadt, Germany), and formic acid from TCI (Tokyo Chemical Industry, Tokyo, Japan). For sample preparation, 10 μl bile was mixed with 40 μl acetonitrile/methanol (1:1) and 1 μl internal standard mix, vortexed, incubated 10 min, centrifuged at 20,000 g for 10 min at 4 °C. Supernatants were transferred to inserts and 10 μl injected into a liquid chromatography with tandem mass spectrometry (LC-MS/MS) apparatus for analysis. LC was performed on a 1290 Infinity System (Agilent Technologies, Santa Clara, CA, USA) with a flow rate of 0.4 ml/min at 50 °C on an Aeris™ PEPTIDE XB-C18 reversed-phase column (1.7 μm, 100 mm × 2.1 mm, Phenomenex®, Torrance, CA, USA). MS/MS was performed on a QTrap 6500 Triple Quadrupole hybrid Ion Trap mass spectrometer (Sciex, Foster City, CA, USA) applying electrospray ionization in both positive and negative modes. Metabolites were separated on a nonlinear gradient over 55 min, the mobile phase consisted of 0.1% formic acid in water and 0.1% formic acid in acetonitrile, as described previously.^32^ Multiple reaction monitoring was used to measure at least two transitions per metabolite. Measurements were normalized using d4-deoxycholate. Metabolite intensities were quantified using MultiQuant™ software version 2.1.1 (Sciex), manually verifying all peaks. The results are shown in Tables S3 and S4.

A previously published method was used for phospholipid preparation.^33^ Briefly, 50 μl ice-cold methanol containing the 12 PC and 20 PC standards was mixed with 5 μl bile, followed by 100 μl chloroform, vortexed for 1 min, and centrifuged at 20,000 g for 10 min at 4 °C. The supernatant was transferred, mixed with 20 μl 50 mM citric acid and 80 μl chloroform, vortexed, and centrifuged for 1 min. A 50-μl sample of the lower organic phase was collected and lyophilized. Phospholipids were reconstituted in 50 μl 2-propanol/acetonitrile/water (2:1:1), incubated 30 min at 25 °C, centrifuged at 15,000 × *g* for 15 min, and 40 μl of the supernatant transferred to inserts; 10 μl was injected as technical replicates using the same LC-MS as for bile acids. The column used was a BEH Shield RP18 (1.7 μm, 130 Å, 150 × 2.1 mm ID, Waters, Milford, MA, USA). Lipids were separated on a 25-min nonlinear gradient with 1:1:1 methanol/acetonitrile/water and 5 mM ammonium acetate and 2-propanol with 5 mM ammonium acetate, as previously described.^33^ Multiple reaction monitoring measured one transition per metabolite in negative mode; 56 PC and 20 PE species were monitored. Measurements were normalized to the mean of 12 PC and 20 PE standards. The results are presented in Table S5.

For liver tissue analysis, donor livers were sampled at organ arrival and after implantation (during IRI) and snap-frozen in liquid nitrogen. Tissues were homogenized with a FastPrep (2 × 60 s, 4.5 m/s) in 0.8 ml 50% acetonitrile/50% methanol. The debris was pelleted by centrifugation (3,000 g, 3 min, 4 °C), and 10 μl of supernatant transferred into a new tube. Subsequently, 40 μl acetonitrile/methanol and 1 μl internal standards (d4-CA, d4-DCA, d4-CDCA) were added. Samples were vortexed, incubated on a rocking platform (1,000 rpm, 10 min, room temperature [RT]), and centrifuged (20,000 g, 10 min, 4 °C). A 45-μl sample of supernatant was transferred to microinjection tubes, and 10 μl injected per LC-MS run.

For serum analysis, a volume of 15 μl was utilized. Subsequently, 60 μl of a buffer comprising 50% acetonitrile and 50% methanol was added, along with 1 μl of the internal bile acid standards. The mixture was vortexed, incubated (1,000 rpm, 10 min, RT), and centrifuged (20,000 × g, 10 min, 4 °C). A 65-μl sample of the supernatant was transferred to new tubes and lyophilized. Before LC-MS analysis, the dried residues were reconstituted in 40 μl of a 1:1 solution of acetonitrile and methanol, vortexed, and briefly sonicated in an ultrasonic water bath. Finally, the samples were transferred to microinjection vials, and 18 μl was injected for each LC-MS run.

Absolute quantification of bile acids in liver tissue and serum was achieved using calibration curves generated for 10 standard analytes ranging from 1 fmol to 100 pmol, measured in technical replicates, based on a serially diluted internal standard mix containing 0.2 mM of each compound in a total volume of 200 μl. The results are presented in Table S10 and S11. All metabolomics data have been deposited in the publicly available repository PeptideAtlas, and can be downloaded from http://www.peptideatlas.org/PASS/PASS05891.

### Quantitative polymerase chain reaction

Quantitative real-time polymerase chain reaction PCR (qPCR) was used to assess mRNA expression bile transporters *ABCB11*, *ABCB4*, *ABCG5/8*, and *CFTR* as well as *CYP7A1* and *CYP8B1* in liver tissue before and after reperfusion. Total RNA was extracted using a Trizol-based protocol with chloroform-based phase separation following the manufacturer’s instructions (RNASolv® Reagent, Omega Bio-tek Inc., Norcross, GA, USA) RNA purity and concentration were determined using a NanoDrop spectrophotometer (Thermo Fisher, Waltham, MA, USA). cDNA was synthesized from 1 μg RNA using the ABScript Kit (RK20400, Abclonal, Woburn, MA, USA). qPCR was performed on a Quantstudio 3 (Applied Biosystems, Thermo Fischer Scientific, Waltham, MA, USA) with SYBR Green chemistry. Primers and the reference gene beta Actin (*ACTB*) were designed with NCBI/Primer-BLAST (National Library of Medicine, Bethesda, MD, USA) and obtained from Eurofins (Eurofins Scientific, Luxembourg, Luxembourg) (Table S2). Relative expression was calculated using the ΔCt, and normalized to *ACTB* expression.

### Bile acid analysis

Biliary bile acids are displayed in both relative (%) and absolute quantities (peak area). Intrahepatic and serum bile acid levels were assessed as part of an exploratory analysis and are reported as pmol/mg and μmol/L, respectively. The abundances of each individual bile acid form are expressed as the sum of their taurine and glycine conjugates to allow for a more comprehensive assessment of their composition. The categorization of bile acids as hydrophobic and hydrophilic is based on their hydrophobicity index (HIx), as described previously.^34^ CA was regarded separately because of its nearly neutral HIx.

### Statistical analysis

Statistical analyses were performed using IBM SPSS Statistics (version 29.0, IBM, Armonk, NY, USA) and illustrated using GraphPad Prism (version 10.2.3, Dotmatics, Boston, MA, USA). Nominal and ordinal data were compared using the Fisher’s exact and Χ^2^ test. The data were tested for normal distribution using the Shapiro–Wilk test. In cases of normal distribution, paired and independent *t* tests were applied to compare the data between and within the groups. If not normally distributed, Mann–Whitney *U* and Wilcoxon tests were used for comparisons. A Spearman correlation was used to analyze the relationship between metabolite levels in bile and serum levels of aspartate aminotransferase (AST) and alanine aminotransferase (ALT). The AUC for total, hydrophobic, and hydrophilic bile acid intensities was calculated using the trapezoidal rule to reflect their cumulative abundance over the first 3 days postoperatively. Logistic and Cox regression models were deployed to investigate the association between cumulative bile acid intensities and clinical outcomes. Because of the relatively small sample size, the analyses were not adjusted for multiple testing.

## Results

### Demographics

The demographics of both groups are displayed in Fig. 1A and in Table S1.

### Biliary bile acid analysis

**Fig. 2:**
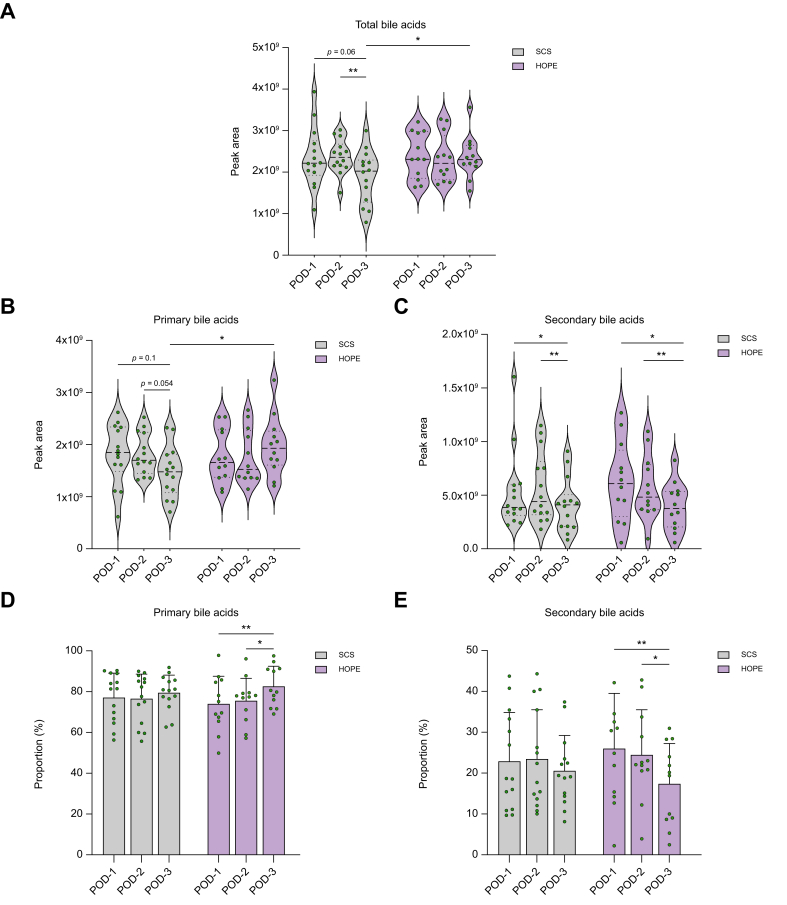
Total bile acids, primary bile acids, and secondary bile acids on POD-1-3. (A) Total bile acids in HOPE *vs.* SCS. (B) Primary bile acids. (C) Secondary bile acids (D,E) Proportions of primary and secondary bile acids. Values of *p* from paired/independent *t* tests or non-parametric tests depending on data distribution; significant values shown (∗*p* ≤0.05, ∗∗*p* ≤0.01). HOPE, hypothermic oxygenated machine perfusion; POD, postoperative day; SCS, static cold storage.

**Fig. 3:**
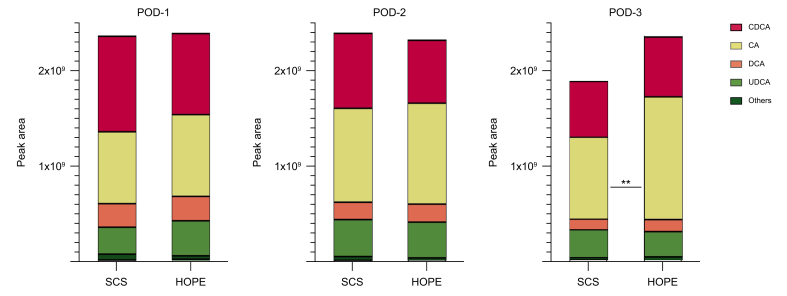
Proportions of most abundant individual bile acids for both groups on POD-1–3. Others include: ursocholic acid, glycohyocholic acid, dehydrocholic acid, tauro-muricholic acids, lithocholic acid. Values of *p* from Mann–Whitney *U* test; ∗∗*p* ≤0.01. CA, cholic acid; CDCA, chenodeoxycholic acid; DCA, deoxycholic acid; HOPE, hypothermic oxygenated machine perfusion; POD, postoperative day; SCS, static cold storage; UDCA, ursodeoxycholic acid.

Nineteen bile acids were detected and quantified across all bile samples. In the SCS group, the amount of biliary bile acid decreased over the 3 days (total bile acids [TBA]: postoperative day [POD]-1 *vs.* POD-3: *p* = 0.06; POD-2 *vs.* POD-3: *p* = 0.006). In contrast, HOPE-treated organs maintained a stable bile acid efflux, resulting in significantly higher bile acid levels on the third day post-LT compared with the control group (SCS TBA POD-3 *vs.* HOPE TBA POD-3: *p* = 0.047) (Fig. 2A). Similarly, the amount of biliary primary bile acids was significantly higher on POD-3 in perfused organs, compared with cold-stored allografts (*p* = 0.027) in whom levels of PBAs tended to decline during the postoperative course (POD-1 *vs.* POD-3: *p* = 0.054) (Fig. 2B). The difference in bile acid levels was predominantly driven by significantly higher quantities of cholic acid in HOPE-treated organs on POD-3 (*p* = 0.006) (Fig. 3). Correspondingly, the proportion of PBAs increased significantly from POD-1 to POD-3 in the HOPE group (POD-1 *vs.* POD-3: *p* = 0.005; POD-2 *vs.* POD-3: *p* = 0.01) but remained unaltered in SCS organs (Fig. 2D). The amount of secreted SBAs declined significantly in both groups (HOPE POD-1 *vs.* POD-3: *p* = 0.004; POD-2 *vs.* POD-3: *p* = 0.28; SCS: POD-1 *vs.* POD-3: *p* = 0.035; POD-2 *vs.* POD-3: *p* = 0.009) (Fig. 2C). The relative abundance of SBAs decreased significantly in HOPE group (POD-1 *vs.* POD-3: *p* = 0.005; POD-2 *vs.* POD-3: *p* = 0.01) while remaining constant in the control group (Fig. 2E).

**Fig. 4:**
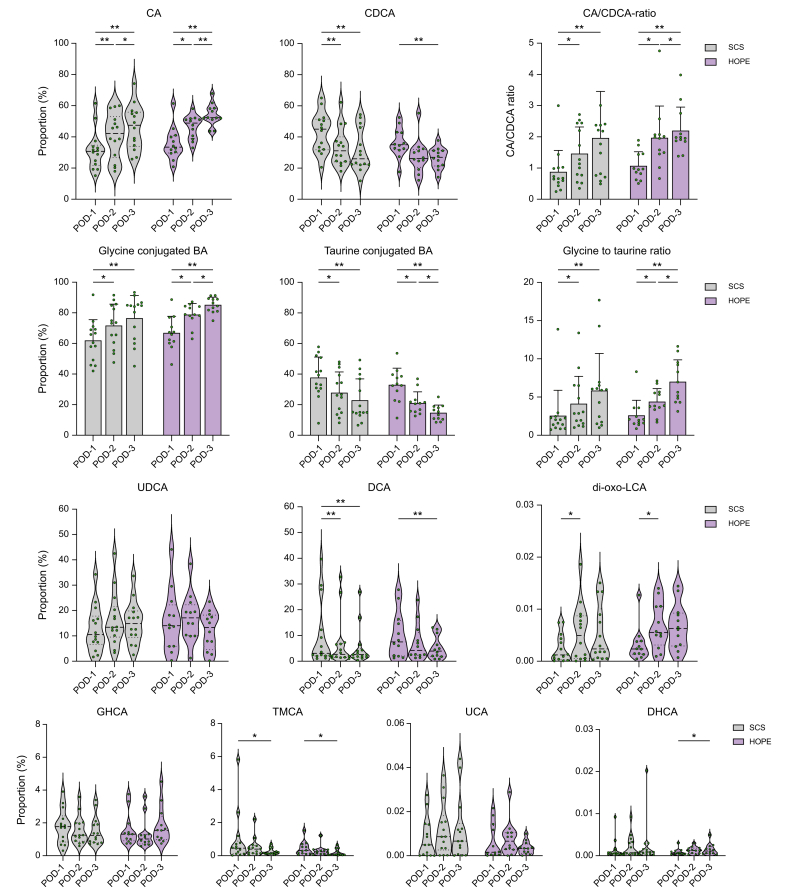
Proportions of individual bile acids for both groups on POD-1–3. Proportions in HOPE *vs.* SCS. Values of *p* from *t* tests or non-parametric tests depending on distribution; ∗*p* ≤0.05, ∗∗*p* ≤0.01. CA, cholic acid; CDCA, chenodeoxycholic acid; DCA, deoxycholic acid; DHCA, dehydrocholic acid; GHCA, glycohyocholic acid; HOPE, hypothermic oxygenated machine perfusion; LCA, lithocholic acid; POD, postoperative day; SCS, static cold storage; TMCA, tauro-muricholic acids; UCA, ursocholic acid; UDCA, ursodeoxycholic acid.

Over the course of the 3 days, allografts from both groups demonstrated a significant increase in cholic acid levels (HOPE POD-1 *vs.* POD-3: *p* <0.001; POD-2 *vs.* POD-3: *p* = 0.006; SCS: POD-1 *vs.* POD-3: *p* <0.001; and POD-2 *vs.* POD-3: *p* = 0.02) and significant decline of CDCA levels (HOPE POD-1 *vs.* POD-2: *p* = 0.006; POD-1 *vs.* POD-3: *p* = 0.06; SCS: POD-1 *vs.* POD-2: *p* = 0.003; and POD-1 *vs.* POD-3: *p* = 0.008). Correspondingly, the CA/CDCA ratio increased significantly from POD-1 to POD-3 for both conservation methods (HOPE POD-1 *vs.* POD-2: *p* = 0.012; POD-1 *vs.* POD-3: *p* = 0.004; SCS: POD-1 *vs.* POD-2: *p* = 0.011; and POD-1 *vs.* POD-3: *p* = 0.008) (Fig. 4). The biliary proportion of DCA (HOPE POD-1 *vs.* POD-3: *p* = 0.004 and SCS: POD-1 *vs.* POD-3: *p* = 0.026) and tauro-muricholic acid (TMCA) (HOPE POD-1 *vs.* POD-3: *p* = 0.023 and SCS: POD-1 *vs.* POD-3: *p* = 0.041) declined significantly in both groups, whereas dehydrocholic Acid (DHCA) increased significantly only in perfused organs (HOPE POD-1 *vs.* POD-3: *p* = 0.041) (Fig. 4). In between the groups, composition of individual bile acids was mostly comparable. Most of the BAs were conjugated to glycine. The ratio of glycine to taurine conjugated BA increased significantly in both groups during the sampling period (HOPE POD-1 *vs.* POD-2: *p* = 0.041; POD-1 *vs.* POD-3: *p* = 0.003; SCS: POD-1 *vs.* POD-2: *p* = 0.019; and POD-1 *vs.* POD-3: *p* = 0.008) (Fig. 4).

**Fig. 5:**
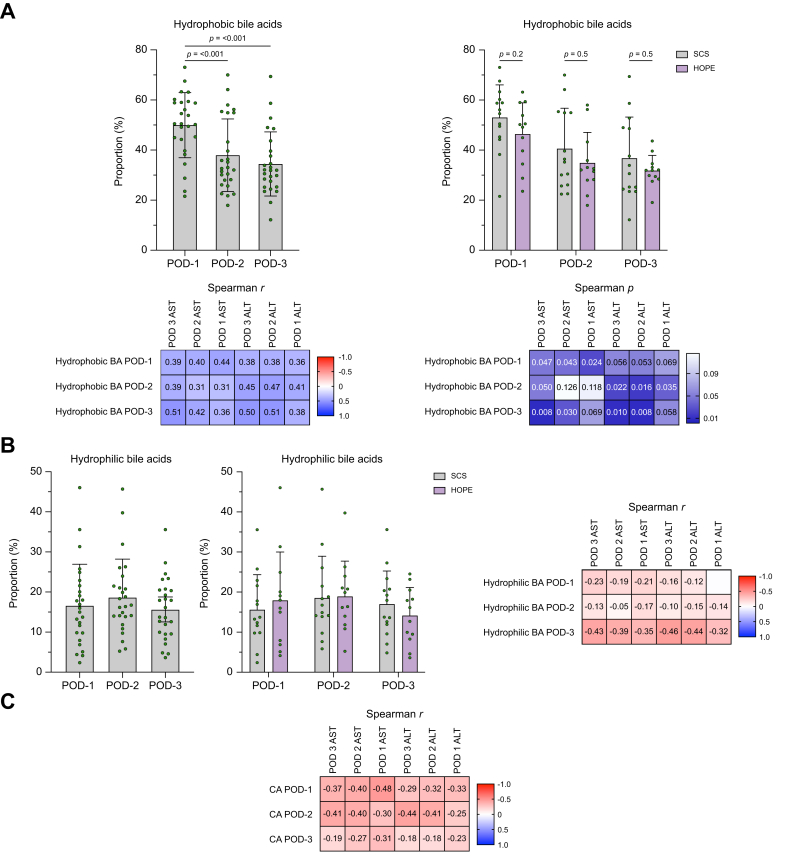
Hydrophobic and hydrophilic bile acids and their correlation with AST and ALT on POD-1, -2, and -3. (A) Hydrophobic bile acids for all patients, HOPE, and SCS group. Values of *p* for paired *t* test, independent *t* test, Mann–Whitney *U* test and Wilcoxon signed-rank test. Correlations to serum AST and ALT levels (Spearman’s *r*, *p*). (B) Hydrophilic bile acids for all patients, HOPE, and SCS group. Correlation to serum AST and ALT (Spearman’s *r*). (C) Cholic acid correlations to serum AST and ALT (Spearman’s *r*). AST, aspartate aminotransferase; ALT, alanine aminotransferase; CA, cholic acid; HOPE, hypothermic oxygenated machine perfusion; POD, postoperative day; SCS, static cold storage.

The fraction of hydrophobic bile acids in the postoperative bile (CDCA, DCA, and LCA) correlated positively with serum levels of AST and ALT on all 3 days and decreased in both groups from POD-1 to POD-3. Perfused organs showed non-significantly lower levels of both hydrophobic forms (Fig. 5A) as well as serum transaminases (Fig. S1). Levels of hydrophilic bile acids (UDCA, ursocholic acid (UCA), DHCA, and glycohyocholic acid [GHCA]) were comparable during the 3 days and correlated negatively with serum levels of AST and ALT on POD-3 (Spearman’s *r* = -0.458, *p* = 0.019; Spearman’s *r* = -0.429, *p* = 0.029) (Fig. 5B). Similarly, CA levels correlated negatively with ALT and AST serum levels on POD-1 and POD-2 (Spearman’s *r* = -0.481, *p* = 0.013; Spearman’s *r* = -0.405, *p* = 0.049) (Fig. 5C). The Heuman Index of the biliary bile acid composition was comparable on all 3 days between both groups (Table S8).

Biliary levels of riboflavin and protoporphyrin IX were similar between the two groups and did not correlate with serum AST and ALT levels.

#### Biliary phospholipids and BA/PC ratio

**Fig. 6:**
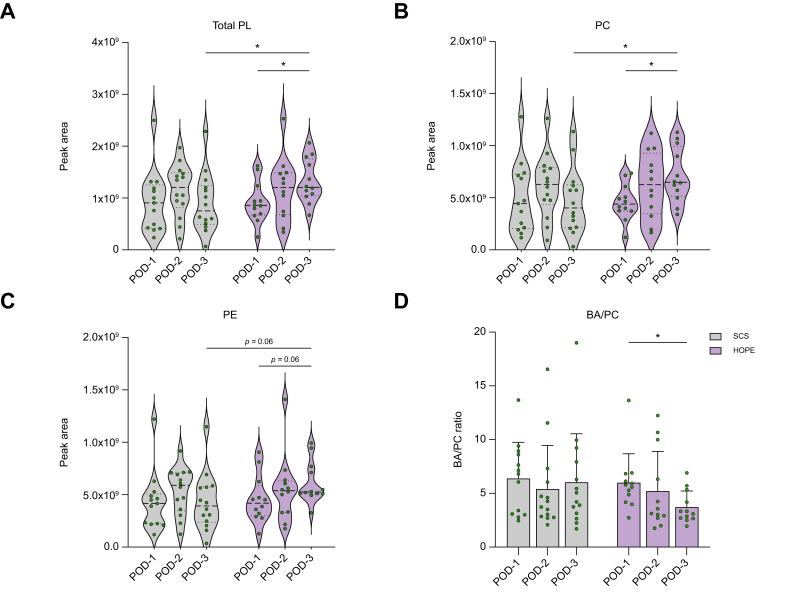
Phospholipids and BA/PC ratio for both groups on POD-1–3. (A–D) Biliary phosphatidylcholine, phosphatidylethanolamine, and bile acid/phosphatidylcholine ratio in HOPE *vs.* SCS. Values of *p* from *t* tests or non-parametric tests; ∗*p* ≤0.05. BA, bile acid; HOPE, hypothermic oxygenated machine perfusion; PC, phosphatidylcholine; PE, phosphatidylethanolamine; PL, phospholipids; POD, postoperative day; SCS, static cold storage.

Total biliary phospholipids levels increased in the HOPE group (POD-1 *vs.* POD-3: *p* = 0.048) through an increase in both PC and PE levels (PC: POD-1 *vs.* POD-3 *p* = 0.037; PE: POD-1 *vs.* POD-3 *p* = 0.06), leading to a significantly higher amounts of total phospholipids (*p* = 0.043) and phosphatidylcholine (*p* = 0.041) on POD-3 in perfused allografts, compared with the control group (Fig. 6A–C). In contrast, the SCS group showed fluctuating biliary phospholipid levels over the postoperative course. While bile acid to PC ratios were comparable between both groups, HOPE-treated allografts exhibited a significant decline in bile acid/PC ratios (POD-1 *vs.* POD-3 *p* = 0.05) (Fig. 6D).

### Hepatic bile transporters and enzymes

**Fig. 7:**
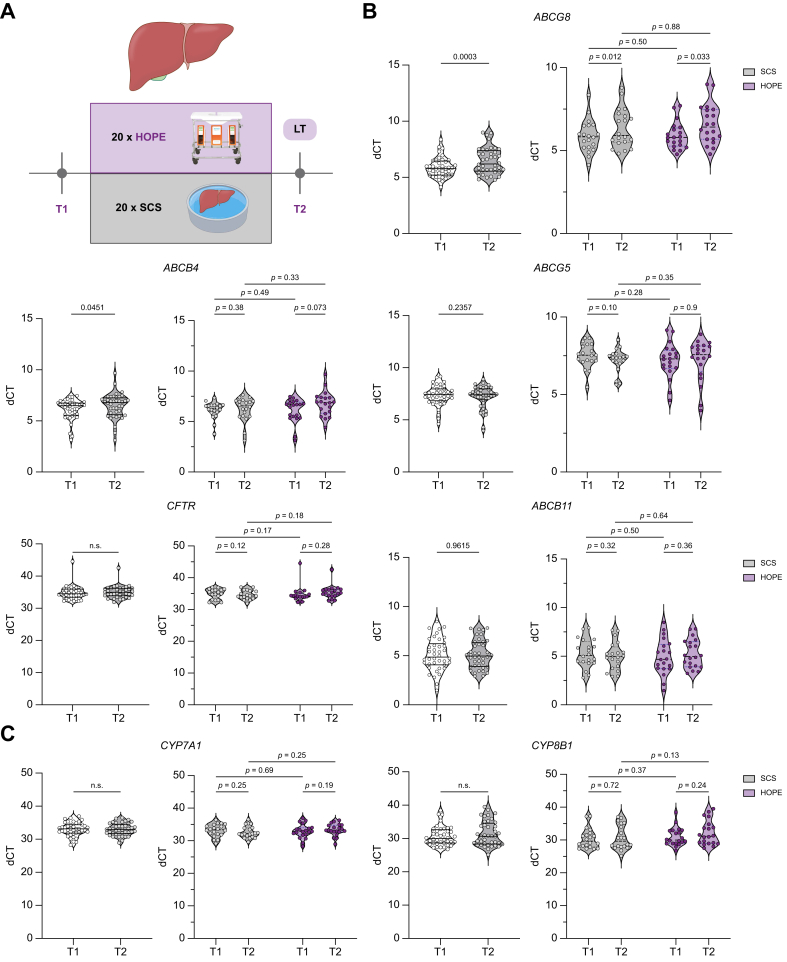
mRNA expression of bile transporters and CYPs before (T1) and after reperfusion (T2) for both groups. (A) Sampling timeline. (B,C) qPCR of ABCB4, ABCG8, ABCB11, ABCG5, CFTR, CYP7A1, and CYP8B1 shown as ΔCT. Values of *p* from *t* tests or non-parametric tests corresponding to data distribution. dCT, delta cycle threshold; HOPE, hypothermic oxygenated machine perfusion; POD, postoperative day; SCS, static cold storage; T, time point.

We conducted qPCR analysis of key proteins involved in bile acid synthesis and bile secretion before (T1) and after reperfusion (T2), aiming to assess the impact of IRI and HOPE on their mRNA expression. mRNA expression of ABCB4, ABCB11, ABCG8/ABCG5, CYP7A1, and CYP8B1 was quantified for 40 patients (HOPE, n = 20; SCS, n = 20). Overall, ABCB4 and ABCG8 were expressed significantly less after reperfusion of the allograft, (ABCB4: T1 *vs.* T2, *p* = 0.045; ABCG5: T1 *vs.* T2, *p* = 0.0003) (Fig. 7B)., Expression of ABCB11, ABCG5, and also CYP7A1 and CYP8B1 was comparable at both timepoints. There were no significant differences in mRNA expression of the bile transporters and enzymes shown between perfused organs and the SCS group (Fig. 7B and C).

### Intrahepatic bile acids

**Fig. 8:**
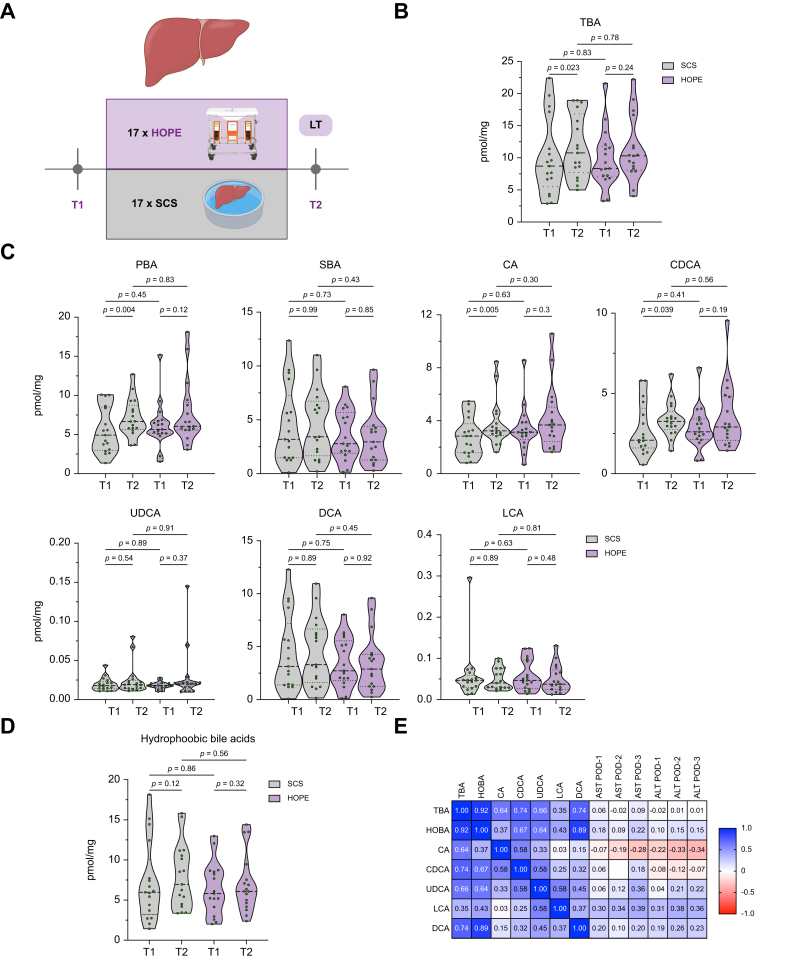
Exploratory LC-MS/MS analysis of bile acid levels in liver biopsies taken before (T1) and after reperfusion (T2). (A) Sampling timeline. (B,C) Intrahepatic bile acids (pmol/mg). Values of *p* for the Wilcoxon signed-rank or Mann Whitney *U* test. (D) Hydrophobic bile acids in pmol/mg. (E) Correlation of postoperative serum AST/ALT with intrahepatic bile acids at T2 (Spearman’s *r*). ALT, alanine aminotransferase; AST, aspartate aminotransferase; CA, cholic acid; CDCA, chenodeoxycholic acid; DCA, Deoxycholic acid; HOBA, hydrophobic bile acids; HOPE, hypothermic oxygenated machine perfusion; LCA, lithocholic acid; POD, postoperative day; PBA, primary bile acids; SBA, secondary bile acids; SCS, static cold storage; TBA, total bile acids; UDCA, ursodeoxycholic acid.

To explore the intrahepatic dynamics of bile acids during organ transplantation, we analyzed bile acid concentrations in liver tissue samples before (T1) and after reperfusion (T2) (Fig. 8A). In the SCS group, intrahepatic bile acids increased significantly following reperfusion (TBA: 8.71 ± 5.96 to 10.77 ± 4.88 pmol/mg; *p* = 0.023), primarily driven by a rise in CA and CDCA (CA: 2.84 ± 1.46 to 3.24 ± 1.77 pmol/mg, *p* = 0.004; CDCA: 2.09 ± 1.61 to 3.2584 ± 1.10 pmol/mg; *p* = 0.039) (Fig. 8B). HOPE-treated organs showed similar intrahepatic bile acid levels at both timepoints. Concentrations of PBAs, SBAs, hydrophobic and individual bile acid species were comparable between groups before and after reperfusion. Intrahepatic bile acids levels after reperfusion did not correlate with serum levels of AST and ALT during the early postoperative phase (Fig. 8B–E).

### Serum bile acid analysis

We performed an exploratory analysis of serum bile acid levels in 16 pair-matched patients from both groups, using blood samples collected at admission (AD) and on POD-1 to -3 (Table S7). In both groups serum bile acid concentrations declined significantly after LT. In the HOPE-treated subgroup, this reduction occurred as early as POD-1 (AD: 165.96 ± 143.95 *vs.* POD-1: 30.61 ± 14.81 μmol/L; *p* = 0.012), whereas it was delayed to POD-2 on the SCS group (AD: 190.46 ± 126.82 *vs.* POD-2: 40.82 ± 58.50 μmol/L *p* = 0.036) (Fig. S2B). Levels of hydrophobic bile acids declined faster in the subgroup of HOPE-treated recipients (AD: 93.99 ± 61.13 *vs.* POD-1: 6.81 ± 4.10 μmol/l; *p* = 0.017) (Fig. S2C) and were lower on POD-1 and POD-2 compared with SCS organs (POD-1: 6.81 ± 4.10 *vs.* 18.93 ± 66.95 μmol/L, *p* = 0.10; POD-2: 4.52 ± 3.78 *vs.* 10.50 ± 19.72 μmol/L, *p* = 0.038). Systemic concentrations of hydrophobic bile acids further correlated with serum AST levels of the corresponding postoperative days (POD-1: Spearman’s *r* = 0.582, *p* = 0.02; POD-2: Spearman’s *r* = 0.695, *p* = 0.004; POD-3: Spearman’s *r* = 0.498, *p* = 0.057) (Fig. S2D).

### Biliary bile acids and clinical outcomes

To investigate the potential association between the early postoperative biliary bile acid composition and clinical outcomes after liver transplantation, we calculated the AUC for hydrophobic, hydrophilic, and TBA intensities using the trapezoidal rule. These values were then analyzed in relation to the incidence of acute cellular rejection and biliary complications within 90 days after LT, and 1-year graft survival via logistic and Cox regression models. No association was observed between cumulative bile acid intensities and the investigated clinical outcomes (Table S6).

## Discussion

We analyzed postoperative bile composition of patients receiving ECD allografts, either treated with HOPE or conserved solely through SCS in the setting of a multicentric, RCT. This study is the first of its kind to investigate the impact of HOPE on bile acid composition and biliary lipid secretion after human LT of extended criteria donation liver allografts.

Using LC-MS/MS, we first identified a biliary bile acid composition consisting of 19 different metabolites and assessed its postoperative dynamic over the course of 3 days postoperatively. This was possible because of the unique institutional protocol of intraoperative biliary T-drain placement, which allows non-invasive postoperative biliary sampling. We found that allografts preserved exclusively through SCS, exhibited a decline in bile acid levels for SBA and PBA forms, while HOPE-treated organs maintained stable bile acid levels through a significant increase in the fraction of PBAs. This dynamic resulted in a significantly higher biliary quantity of bile acids in the HOPE group on POD-3, primarily driven by elevated CA levels. In an exploratory approach, we expanded the bile acid analysis to liver and serum samples, which revealed an intrahepatic accumulation of bile acids after reperfusion as well as slower normalization of serum bile acids levels in patients receiving cold-stored organs.

PBAs are synthesized by the hepatocytes through the sequential activity of cytochrome P450 enzymes and secreted into the intrahepatic canaliculi by ATP-dependent ABC-transporters, most prominently the bile salt export pump (BSEP)/ABCB11.^6^^,^^35^ The expression of both enzymes and transporters is downregulated by IRI,^20^ resulting in an impaired bile acid secretion after LT. Bile acid secretion, and alongside with it bile flow, recover during the first 3 weeks after LT.^36^ Allografts meeting ECD criteria are known to have a slower recuperation of bile acid secretion compared with organs from standard donors.^37^ The findings indicate that HOPE treatment improves the recovery of bile acid efflux in livers from ECD post-transplantation. The relative increase in biliary PBAs for HOPE-treated allografts suggests that this might be as a result of a faster restoration of hepatic bile acid synthesis, particularly of CA. These results may be explained by the mitigation of hepatic IRI and restoration of intracellular ATP reserves as known treatment effects of HOPE^22^^,^^38^ and crucial elements for hepatic bile acid metabolism and secretion.^6^^,^^20^^,^^35^ Preclinical findings parallel the here shown enhancing effect of HOPE on biliary lipid secretion,^39^ implicating a preventive effect of HOPE on cholestasis and accumulation of cytotoxic bile acids after LT.^16^ Notably, since the analyzed liver tissue contained bile canaliculi and ductal elements, the measured bile acids predominantly reflected the biliary system, and true intrahepatocellular accumulation could not be specifically captured.

Furthermore, we showed that HOPE-treated organs exhibit an increase in biliary phospholipids, leading to significantly higher total phospholipids and PC levels on POD-3, compared with allografts in the SCS group. This resulted in a significant decrease in the biliary bile acid/PC ratio for perfused allografts. PC is the principal biliary phospholipid and is secreted into the bile by ABCB4.^35^ It reduces bile acid toxicity by forming mixed micelles with hydrophobic bile acids species, thereby neutralizing their detergents effects.^14^^,^^40^ Phospholipid secretion is also impaired by IRI post-transplantation and may recover slower than bile acid secretion, resulting in an elevated bile acid/PC ratio in early bile after LT.^41^ This disbalance of biliary lipids has been shown to induce bile duct injury^17^^,^^18^^,^^41^ and has been associated with the development of biliary strictures.^19^ Our results suggest that HOPE treatment improves efflux of biliary phospholipids, thereby reducing the bile acid/PC ratio in early postoperative bile after LT. Given the established role of PC in mitigating bile acid toxicity through micelle formation and reduced cell membrane interaction, this suggests a protective effect of HOPE on early postoperative bile composition. Previous clinical studies have shown that HOPE treatment reduces bile duct injury^42^^,^^43^ and incidence of biliary strictures after LT.^26^ Our findings offer an additional mechanistic explanation for these effects. However, although previous studies have outlined an association between the biliary bile acid/PC ratio and the development of biliary strictures^19^ this relationship was not directly investigated in our study. Further research is needed to validate this mechanistic link in bile samples from HOPE-treated LT recipients.

Next, we noted that the postoperative biliary BA pool undergoes substantial changes during the first 3 days after LT, showing a significant shift towards CA and a decline in CDCA and DCA for all allografts. A similar pattern has been reported in previous studies.^36^^,^^44^ The hepatotoxicity of bile acids increases with their hydrophobicity, making CDCA and DCA more cytotoxic than CA.^14^ This was reflected by the positive correlation between biliary and blood levels of hydrophobic BAs and serum levels of AST and ALT. Interestingly, CA exhibited a negative correlation with transaminase levels. These findings suggest that the biliary bile acid pool after LT shifts towards a less cytotoxic composition, which is associated with lower IRI. The synthesis of CA and CDCA as the major PBAs occurs through the alternative (10%) or classical pathway (90%).^6^ While the alternative pathway exclusively synthesizes CDCA, the classical pathway produces both types of BA, with CYP8B1 serving as the key enzyme for CA production.^45^ A recent study demonstrated that IRI during LT induces a reprogramming of PBA synthesis in rodents leading to an increase of TβMCA, which resulted in lower inflammatory injury through inhibition of macrophages by the bile acid metabolite.^20^ Similarly, a cytoprotective adaption of bile acid synthesis has been shown in rodent models for cholestasis and partial hepatectomy.^46^^,^^47^ The observed shift towards an increase in biliary CA levels and its association with lower markers for hepatic injury, suggests that an adaptive protective mechanism may also occur in human bile acid metabolism after LT. Importantly, this adaptive shift in CDCA/CA ratio was observed irrespectively of HOPE treatment. Of note, both groups exhibited high proportions of UDCA in early postoperative bile, likely reflecting its routine postoperative administration. UDCA is a hydrophilic bile acid with well-documented hepatoprotective properties, including reduced cytotoxicity of hydrophobic bile acids.^15^ While prophylactic UDCA administration has been associated with a lower risk of biliary complications in some studies,^48^ our regression analysis did not identify a significant association between biliary levels hydrophilic bile acids, such as UDCA, and clinical outcomes.

Our qPCR analysis of hepatic bile transporters revealed a significant reduction in mRNA expression levels of ABCB4 and ABCG5 following organ reperfusion, compared with before reperfusion. These findings align with prior studies indicating impaired biliary transporters expression as a consequence of IRI.^17^^,^^20^ Interestingly, the expression of the BSEP ABCB11 remained unchanged. This supports the hypothesis that phosphatidylcholine secretion is more affected after LT than bile acid secretion, leading to a higher bile acid/PC ratio and subsequent bile duct injury post-transplantation.^17^^,^^19^^,^^49^ The expression of bile transporters as well as CYP7A1 and CYP8B1 appeared unaffected by HOPE treatment, as no differences between the SCS group and perfused organs were observed at both timepoints. However, as T2 biopsies were acquired 30–60 min after reperfusion, and significant changes in hepatocyte mRNA expression usually occur 4–6 h after induction,^50^^,^^51^ the full extent of potential differences in transporter and enzyme expression between preservation groups may not have been fully captured.

The objective of this study was to examine the impact of HOPE on bile and bile acid composition in patients undergoing LT. Although this study included unique samples, it has several limitations. First, this study centers on the bile analysis of 26 patients, which may limit the generalizability of the findings because of the relatively small sample size. Second, biliary lipids were assessed in relative metabolite quantities, rather than absolute concentrations, therefore being limited regarding its interpretability on actual biological differences and mechanistic relevance. Furthermore, comparisons of measured intensities between different metabolite species may not be directly valid as a result of differences in ionization efficiencies. To address these analytical constraints, absolute metabolite concentrations were determined in the exploratory analysis of liver tissue and serum samples. Thirdly, the 3-day observation period limits the ability to capture changes beyond the third postoperative day and thus provides no information about long-term developments. Finally, the results of this study remain primarily descriptive, as no correlations were established between bile acid measurements and clinical outcomes.

Despite these limitations, this study provides unique evidence supporting the protective effect of HOPE on bile composition of ECD organs as well as compelling insights into post-LT changes in BA metabolism. HOPE-treated organs showed a faster recuperation of bile acid and phospholipid efflux after LT. Through a comprehensive bile acid analysis, we identified significant postoperative changes in the bile acid pool towards a composition associated with reduced cytotoxicity and hepatocellular damage. Further research is necessary to explore the clinical relevance of postoperative bile composition and its interaction with IRI, particularly regarding biliary complications in liver allografts from donors after circulatory death.

## Abbreviations

AD, admission; ALT, alanine aminotransferase; AST, aspartate aminotransferase; BA/PC, bile acid to phosphatidylcholin ratio; BSEP (ABCB11), bile salt export pump; CA, cholic acid; CDCA, chenodeoxycholic acid; DBD, donation after brain death; DCA, deoxycholic acid; DHCA, dehydrocholic acid; DLPC, 1,2-dilauroyl-sn-glycero 3 phosphocholine 12:0 PC; ECD, extended criteria donation; GDCA, glycodeocycholic acid; GHCA, glycohyocholic acid; GUDCA, glucoursodeoxycholic acid; HIx, hydrophobicity index; HOPE, hypothermic oxygenated machine perfusion; IRI, ischemia–reperfusion injury; LCA, lithocholic acid; LC-MS/MS, liquid chromatography-tandem mass spectrometry; LT, liver transplantation; NAS, non-anastomotic strictures; PBA, primary bile acid; PC, phosphatidylcholine; PE, phosphatidylethanolamine; POD, postoperative day; qPCR, quantitative polymerase chain reaction; RCT, randomized controlled trial; SBA, secondary bile acid; SCS, static cold storage; T, time point; TBA, total bile acids; TCA, taurocholic acid; TCDCA, taurochenodeoxycholic acid; TMCAs, tauro-muricholic acids; TUDCA, tauroursodeoxycholic acid; UCA, ursocholic acid; UDCA, ursodeoxycholic acid.

## Authors’ contributions

Conceptualization: FS, GL. Methodology: FS, DU, MK, DM. Data curation: FS, DU, IL, ZC, DM. Formal analysis: FS. Investigation: FS, ZC, MK, DM. Validation: FS, DU, IL. Writing – original draft: FS. Writing – review and editing: IL, JE, FT, PS, MK, DM, GL. Project administration: IL, GL. Funding acquisition: ZC, GL. Resources: ZC, JE, FT, PS, JP, PH, UPN, CE, CM, PD, DM, GL. Supervision: GL. Critical appraisal and review of the manuscript: all authors.

## Data availability

LC-MS/MS results were uploaded to PeptideAtlas (http://www.peptideatlas.org/PASS/PASS05891). Pseudonymized patient data will be provided upon reasonable request to the corresponding author. Should a Letter to the Editor be submitted, reviewers will receive password-protected access, with data becoming publicly available upon publication.

## Financial support

This research project was supported by the START-Program (#136/17 to Georg). The study was furthermore funded by the 10.13039/501100001659Deutsche Forschungsgemeinschaft (LU 2185/2-1). This work is supported by the 10.13039/501100001659German Research Foundation (10.13039/501100001659DFG 10.13039/501100003383CRC/TR 412, Project-ID 535081457, and SFB1382, Project-ID 403224013) and under Germany's Excellence Strategy – EXC 3118/1 – project number 533770413. PS is supported by the 10.13039/501100001659German Research Foundation grant STR1095/6-1 (Heisenberg professorship).

## Conflicts of interest

GL reports receiving research funding and speakers’ fees from Astellas Pharma, XVIVO, Bridge to Life, Organ recovery systems, Wyss Liver4Life, Orphalan, and Aferetica S.R.L, and is on the advisory board of OrganOx, outside the submitted work. CE reports receiving an Else Kröner Fresenius Excellence Scholarship (German Research Foundation, DFG) and an EU-Horizon grant, has shares with UCL spin-off company Hepyx Ltd, has received consulting fees from and is on the advisory board of Albireo/Ipsen and Boehringer Ingelheim, and has received lecture honoraria/travel support from Gilead and Albireo/Ipsen. FT reports research funding to his institution from AstraZeneca, MSD, Gilead, and Agomab, and fees for consulting or lectures from AstraZeneca, Gilead, GSK, Abbvie, Alnylam, BMS, Pfizer, Madrigal, Novartis, Novo Nordisk, MSD, and Sanofi. PS reports receiving grants and honoraria from Arrowhead Pharmaceuticals, CSL Behring, and Grifols Inc., consulting fees or honoraria from AiRNA Pharmaceuticals, Alnylam Pharmaceuticals, Arrowhead Pharmaceuticals, BioMarin Pharmaceutical, BridgeBio/Gondola, Dicerna Pharmaceuticals, GSK, IPSEN, Intellia Pharmaceuticals, Takeda Pharmaceuticals, Novo Nordisk, Wave Pharmaceuticals, and Ono Pharmaceuticals, participating in leadership or fiduciary roles in Alpha1-Deutschland, Alpha1 Global, and material transfer support for Vertex Pharmaceuticals and Dicerna Pharmaceuticals.

Please refer to the accompanying ICMJE disclosure forms for further details.
