# Supplementary material for: Effects of hypothermic oxygenated machine perfusion on bile composition after liver transplantation – Findings from a randomized controlled trial
Source: JHEP Rep. 2025 Oct 17;8(2):101647. doi: 10.1016/j.jhepr.2025.101647 (PMC12810548; doi:10.1016/j.jhepr.2025.101647)
Supplement: Multimedia component 1 [file mmc1.pdf]

Effects of hypothermic oxygenated machine perfusion on bile composition after liver transplantation – Findings from a randomized controlled trial

Frederik Schliephake, Isabella Lurje, Deniz Uluk, Janina Eden, Zoltan Czigany, Justus Pein, Peri Husen, Cornelius Engelmann, Christoph Michalski, Marlene Kohlhepp, Pavel Strnad, Philipp Dutkowski, Frank Tacke, Ulf Peter Neumann, David Meierhofer, Georg Lurje

Table of contents

Fig. S1……………………………………………………………………………….2

Fig. S2……………………………………………………………………………….3

Table S1…………………………………………………………………………...5

Table S2…………………………………………………………………………...6

Table S3…………………………………………………………………………...7

Table S4…………………………………………………………………………...8

Table S5…………………………………………………………………………...9

Table S6……………………………………………………………………………10

Table S7……………………………………………………………………………11

Table S8……………………………………………………………………………12

Table S9……………………………………………………………………………13

Table S10………………………………………………………………………...14

Table S11………………………………………………………………………...15

Fig. S1 Serum ALT und AST levels of patients of the HOPE and SCS group included in the subgroup analysis.

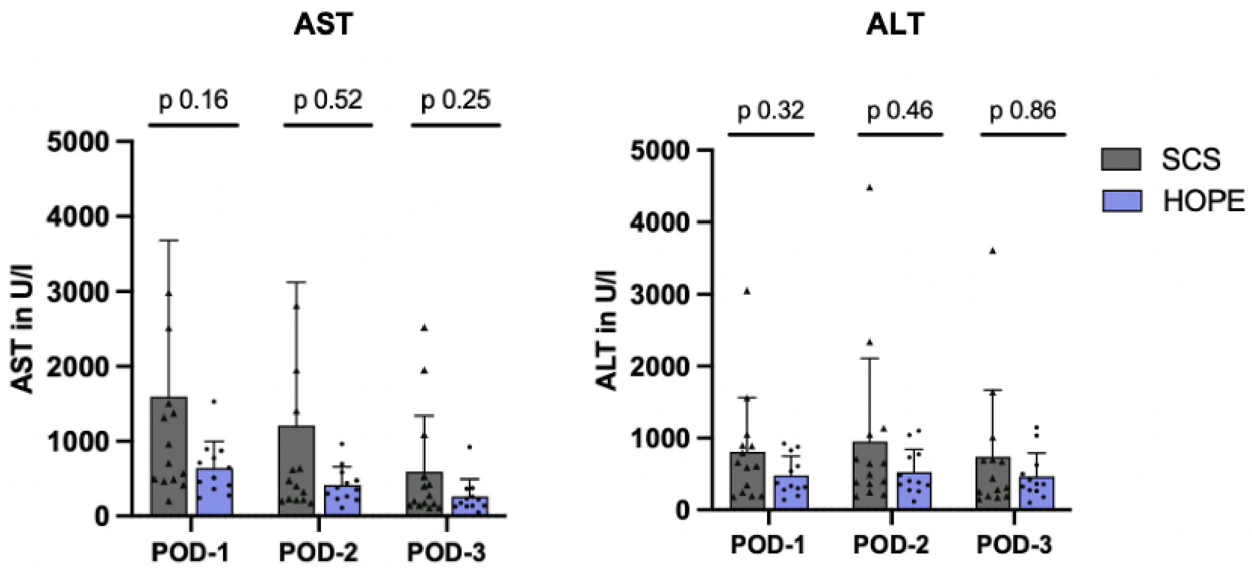

Mann-Whitney U Test was used for the comparison of both groups

ALT (Alanine Aminotransferase); AST (Aspartate Aminotransferase); HOPE hypothermic, oxygenated machine perfusion; SCS Static cold storage

Fig. S2 Exploratory LC-MS/MS analysis of bile acid levels in serum samples acquired at patient admission and on the postoperative days 1-3

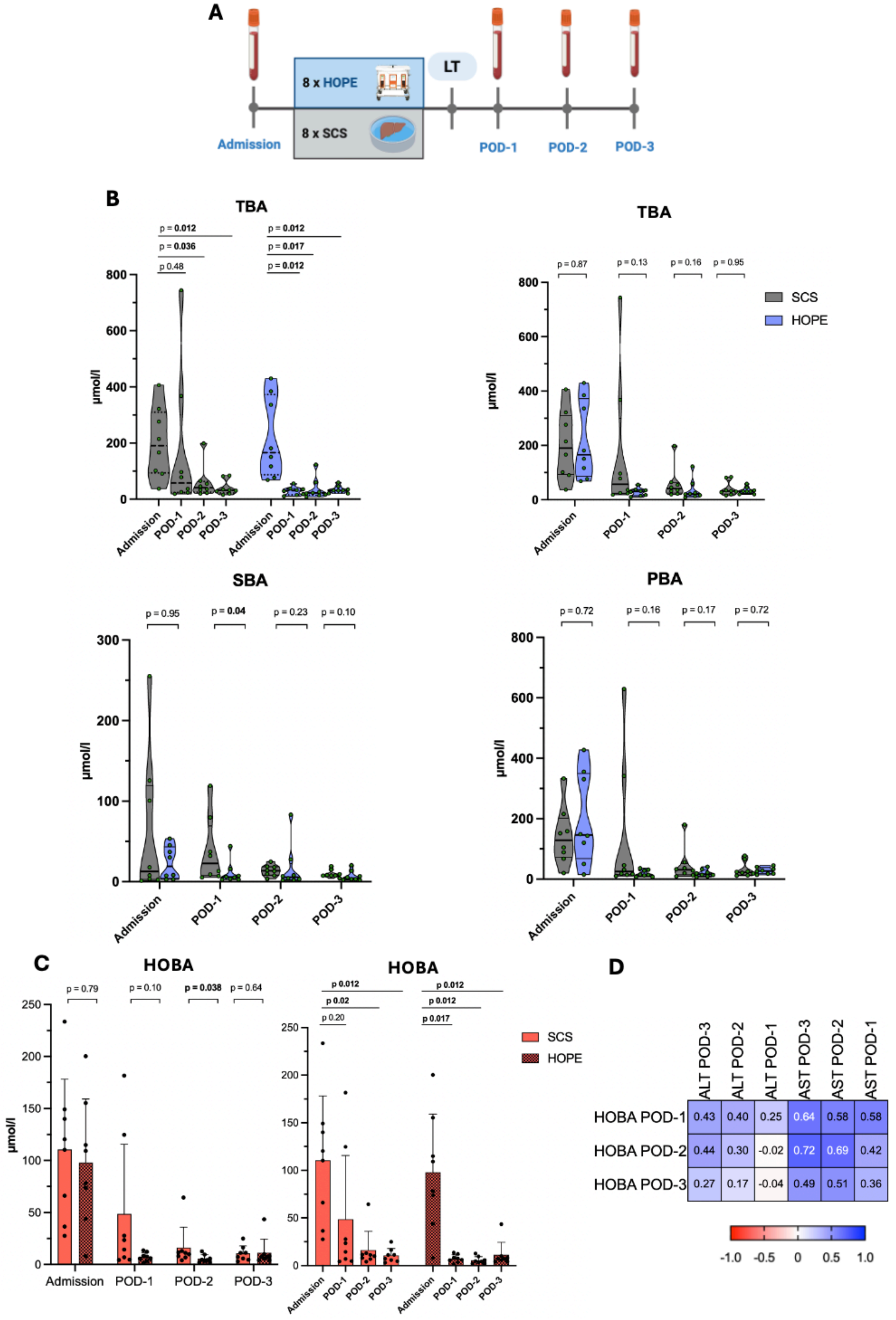

(A) Sampling timeline of patient serum samples (B-C) Serum bile acid levels in µmol/l determined through LC-MS/MS analysis of blood samples acquired according to A. The Mann–Whitney U test and Wilcoxon signed-rank test were used for comparisons between and within groups, respectively. (D) Spearman r of serum AST and ALT levels correlated with serum levels of hydrophobic bile acids on corresponding postoperative days.

ALT (Alanine Aminotransferase); AST (Aspartate Aminotransferase); HOPE hypothermic, oxygenated machine perfusion; HOBA Hydrophobic bile acids; PBA primary bile acids; POD postoperative day; SBA secondary bile acids; SCS Static cold storage, TBA Total bile acids

Table S1 Demographics, donor specific information, intraoperative and preservation characteristics of patients included in the bile analysis

Characteristics	All patients (n = 26)	SCS (n = 14)	HOPE (n = 12)	*p*
Donor Characteristics				
Age, y, median (IQR)	70 (61 – 77)	66 (58 – 79)	73 (68 – 75)	0.840
BMI, kg/m^2, median (IQR)	28 (24 – 39)	28 (25 – 30)	27 (24 – 29)	0.882
Sex, female: male, n (%)	12 (46%): 14 (54%)	6 (43%): 8 (57%)	6 (50%): 6 (50%)	0.716
ET-DRI Score*	2.040 (1.875 – 2.091)	2.070 (1.913 – 2.091)	1.960 (1.880 – 2.060)	0.993
Cause of death n (%)				
Cerebrovascular accident	14 (54%)	9 (64%)	5 (42%)	0.431
Anoxia	8 (31%)	2 (14%)	6 (50%)	0.09
Trauma	4 (15%)	3 (22%)	1 (8 %)	0.598
ECD-Criteria				
Donor Resuscitation, yes, n (%)	9 (36%)	4 (29%)	5 (46%)	0.434
Donor age ≥65	16 (62%)	7 (50%)	9 (75%)	0.248
Donor ICU ≥7 days	6 (23%)	2 (14%)	4 (33%)	0.356
Donor BMI >30 kg/m^2	6 (23%)	3 (21%)	3 (25%)	>0.999
Macro/mixed steatosis >40%	6 (24%)	3 (23%)	3 (25%)	>0.999
Serum sodium >165 mmol/L	1 (4%)	1 (7%)	0 (0%)	>0.999
Serum AST/ALT >3× of normal	9 (35%)	6 (43%)	3 (25%)	0.429
Serum bilirubin >2 mg/dL	2 (8%)	1 (7%)	1 (8%)	>0.999
No. of fulfilled ECD-Criteria				
1 Of 7	10 (39%)	6 (43%)	4 (33%)	0.701
2–3 Of 7	16 (61%)	8 (57%)	8 (67%)	0.701
>3 Of 7	0	0	0	n/a
Microsteatosis, n (%)				
None to mild (0–29%)	16 (76%)	7 (70%)	9 (82%)	0.635
Moderate to severe (≥30%)	5 (24%)	3 (30%)	2 (18%)	0.635
Macrosteatosis, n (%)				
None to mild (0–29%)	21 (91%)	11 (92%)	10 (91%)	>0.999
Moderate to severe (≥30%)	2 (9%)	1 (8%)	1 (9%)	>0.999
Graft weight, g, median (IQR)	1643 (1408–1970)	1690 (1466–1819)	1591 (1346–1819)	0.949
Recipient Characteristics				
Age, y, median (IQR)	61 (56–65)	62 (54–65)	61 (58–64)	0.543
BMI, kg/m^2, median (IQR)	28 (26–31)	28 (26–28)	28 (24–31)	0.395
Sex, female: male, n (%)	4 (15%): 22 (85%)	2 (14%): 12 (86%)	2 (17%): 10 (83%)	>0.999
LabMELD, median (IQR)	14 (9–25)	18 (8–26)	10 (8–14)	0.170
BAR Score, median (IQR)§	6 (5–11)	7 (5–13)	5 (4–10)	0.354
Listing indications, n (%)				
Alcoholic cirrhosis	9 (35%)	5 (36%)	4 (33%)	>0.999
MASH cirrhosis	7 (27%)	4 (29%)	3 (25%)	>0.999
Viral cirrhosis	5 (19,2%)	2 (14%)	3 (25%)	0.635
HCC in cirrhosis	15 (57,7%)	7 (50%)	8 (66,7%)	0.453
PSC/PBC	2 (7,7%)	1 (7,1%)	1 (8,3%)	>0.999
Other	8 (30,8%)	6 (43%)	2 (17%)	0.216
Preservation Details				
Total preservation time, min, median (IQR)	523 (472–606)	538 (476–596)	523 (473–612)	0.920
Cold preservation time before HOPE, min, median (IQR)	386 (320–465)	NA	386 (320–465)	NA
Machine perfusion time, min, median (IQR)	136 (105–180)	NA	136 (105–180)	NA
Warm ischemic, min, median (IQR)	49 (39–55)	49 (40–53)	49 (39–56)	0.866

Data presented as median and interquartile range if not noted otherwise.
† Mann-Whitney U Test was used for continuous variables, Chi Square Test or two-sided Fisher's exact test for categorical variables.
Abbreviations: ALT alanine aminotransferase, AST aspartate aminotransferase, BAR Balance of Risk Score, BMI body mass index, ECD extended criteria donor, ET-DRI Eurotransplant Donor Risk Index, HCC hepatocellular carcinoma, HOPE Hypothermic, oxygenated machine perfusion; ICU intensive care unit, IQR interquartile range, LabMELD laboratory Model for End-Stage Liver Disease, MASH metabolic dysfunction–associated steatohepatitis; PSC primary sclerosing cholangitis, PBC, primary biliary cholangitis, SCS Static cold storage

Table S2 Primer pairs used for qPCR in liver tissue

Target	Sequence Forward (3' -> 5')	Sequence Reverse (5' -> 3')
ABCB4	CAGTACTGGTGCACTTTCTACAAGACTT	TGCAATTAAAGCCAACCTGGTT
ABCB11	GTCGGACCTGCATTGTCATTG	ATGTGTGTCTGAGATTCTTGCATT
ABCG5	CAAGTCCCAGTCCTGCTGTC	GTAGGAGGCATGGAGGATGC
ABCG8	GCAGCAGCTGGGTCTAAGAG	CAGCTGCTCAAACCAAGGGA
CFTR	GAGGCAGTCTGTCCTGAACC	GAGCCACAGCACAACCAAAG
CYP7A1	AGCATTGACCCGATGGATGG	CATTGTGCGCAGTCCTGAAC
CYP8B1	CCTCTTTCTGGGCCCTCTTG	GGGTCAACATGGGGTAGTGG
ACTB	TCACCCACACTGTGCCCATCTACGA	CAGCGGAACCGCTCATTGCCAATGG

Table S3 Biliary Intensities in Peak Area (PA) of individual bile acids

	SCS			HOPE		
**POD-**	**1**	**2**	**3**	**1**	**2**	**3**
**CDCA**						
**MEDIAN**	1.04E+09	7.75E+08	5.30E+08	8.65E+08	5.18E+08	6.85E+08
**IQR1**	8.13E+08	6.29E+08	3.80E+08	6.87E+08	4.21E+08	5.45E+08
**IQR3**	1.15E+09	9.05E+08	7.76E+08	1.04E+09	1.02E+09	7.24E+08
**CA**						
**MEDIAN**	6.78E+08	9.84E+08	7.94E+08	6.92E+08	1.01E+09	1.23E+09
**IQR1**	5.40E+08	8.28E+08	6.04E+08	6.40E+08	8.88E+08	1.07E+09
**IQR3**	1.00E+09	1.13E+09	1.05E+09	1.10E+09	1.20E+09	1.40E+09
**DCA**						
**MEDIAN**	7.58E+07	5.73E+07	4.57E+07	1.57E+08	9.36E+07	7.82E+07
**IQR1**	5.34E+07	3.36E+07	2.43E+07	6.28E+07	5.73E+07	4.94E+07
**IQR3**	1.96E+08	1.70E+08	8.94E+07	2.88E+08	2.07E+08	2.40E+08
**UDCA**						
**MEDIAN**	2.94E+08	3.44E+08	2.40E+08	3.98E+08	3.33E+08	2.64E+08
**IQR1**	1.93E+08	2.10E+08	1.61E+08	1.72E+08	3.01E+08	1.53E+08
**IQR3**	3.49E+08	5.10E+08	4.13E+08	4.29E+08	4.65E+08	4.01E+08
**LCA**						
**MEDIAN**	3.33E+04	1.09E+05	4.29E+04	5.27E+04	1.38E+05	1.56E+05
**IQR1**	1.05E+04	1.78E+04	1.69E+04	2.77E+04	8.23E+04	9.51E+04
**IQR3**	8.48E+04	1.95E+05	1.74E+05	7.84E+04	2.19E+05	1.97E+05
**TMA**						
**MEDIAN**	1.12E+07	7.90E+06	3.34E+06	7.53E+06	4.64E+06	2.00E+06
**IQR1**	3.56E+06	3.63E+06	2.14E+06	2.57E+06	2.36E+06	1.22E+06
**IQR3**	2.00E+07	1.40E+07	7.55E+06	1.55E+07	8.15E+06	7.75E+06
**GHCA**						
**MEDIAN**	4.28E+07	3.42E+07	2.42E+07	2.92E+07	2.23E+07	3.48E+07
**IQR1**	2.48E+07	2.15E+07	1.67E+07	2.31E+07	1.66E+07	2.35E+07
**IQR3**	5.57E+07	4.73E+07	3.57E+07	3.89E+07	3.07E+07	6.69E+07
**DHCA**						
**MEDIAN**	1.22E+04	1.79E+04	1.83E+04	1.31E+04	2.91E+04	2.36E+04
**IQR1**	9.87E+03	1.04E+04	8.01E+03	9.95E+03	1.31E+04	1.22E+04
**IQR3**	2.49E+04	8.26E+04	4.00E+04	1.99E+04	3.62E+04	3.85E+04
**UCA**						
**MEDIAN**	1.06E+05	2.23E+05	1.41E+05	3.31E+04	1.30E+05	7.79E+04
**IQR1**	4.39E+03	9.10E+04	2.45E+04	1.34E+04	7.61E+04	3.51E+04
**IQR3**	2.84E+05	3.20E+05	3.39E+05	2.29E+05	2.23E+05	8.93E+04
**TAURINE CONJUGATES**						
**MEDIAN**	9.64E+08	6.39E+08	3.41E+08	8.23E+08	4.03E+08	3.27E+08
**IQR1**	7.28E+08	4.62E+08	2.73E+08	6.36E+08	3.55E+08	2.39E+08
**IQR3**	1.05E+09	8.35E+08	5.11E+08	9.74E+08	5.71E+08	4.06E+08
**GLYCINE CONJUGATES**						
**MEDIAN**	1.31E+09	1.61E+09	1.34E+09	1.45E+09	1.69E+09	1.99E+09
**IQR1**	1.10E+09	1.48E+09	1.01E+09	1.27E+09	1.51E+09	1.81E+09
**IQR3**	1.80E+09	1.97E+09	1.87E+09	2.13E+09	2.06E+09	2.15E+09

Abbreviations: CA *Cholic acid*; CDCA *Chenodeoxycholic acid*; DCA *Deoxycholic acid*; DHCA *Dehydrocholic acid*; GHCA *Glycohyocholic acid*; HOPE *Hypothermic oxygenated machine perfusion*; LCA *Lithocholic acid*; POD *Postoperative day*; SCS *Static cold storage*; TMCA *Tauro-muricholic acids*; UCA *Ursocholic acid*; UDCA *Ursodeoxycholic acid*

Table S4 Biliary proportions of individual bile acids in %

	SCS			HOPE		
**POD**	**1**	**2**	**3**	**1**	**2**	**3**
**CDCA**						
**MEDIAN**	45.05	31.13	25.99	34.97	26.18	26.96
**IQR1**	32.39	24.40	22.25	32.28	23.23	21.83
**IQR3**	50.92	37.06	43.31	42.82	31.34	31.21
**CA**						
**MEDIAN**	30.71	42.22	47.47	33.40	48.83	52.29
**IQR1**	23.10	30.62	35.36	30.17	40.12	51.70
**IQR3**	33.55	50.48	55.38	40.18	51.18	58.23
**DCA**						
**MEDIAN**	3.01	2.64	2.53	7.49	4.18	3.95
**IQR1**	2.08	1.57	1.67	2.16	2.47	1.99
**IQR3**	11.43	6.60	4.18	15.01	9.10	7.52
**UDCA**						
**MEDIAN**	10.51	13.39	14.85	14.03	17.10	13.35
**IQR1**	7.54	10.65	10.44	5.91	10.23	6.72
**IQR3**	16.55	19.65	18.89	19.53	20.42	17.66
**LCA**						
**MEDIAN**	0.0012	0.0045	0.0023	0.0023	0.0055	0.0063
**IQR1**	0.0004	0.0006	0.0009	0.0014	0.0042	0.0031
**IQR3**	0.0041	0.008	0.0086	0.0038	0.0105	0.0090
**TMA**						
**MEDIAN**	0.46	0.4	0.18	0.31	0.24	0.08
**IQR1**	0.15	0.16	0.13	0.14	0.10	0.06
**IQR3**	0.76	0.57	0.37	0.57	0.34	0.30
**GHCA**						
**MEDIAN**	1.77	1.25	1.24	1.31	1.02	1.55
**IQR1**	0.95	0.96	0.83	0.97	0.8	1.05
**IQR3**	2.17	1.98	1.90	1.56	1.27	2.04
**DHCA**						
**MEDIAN**	0.0005	0.0006	0.0009	0.0006	0.0012	0.0011
**IQR1**	0.0003	0.0003	0.0004	0.0004	0.0006	0.0006
**IQR3**	0.0011	0.0032	0.0015	0.0010	0.0018	0.0018
**UCA**						
**MEDIAN**	0.0049	0.0086	0.0066	0.00158	0.0065	0.0032
**IQR1**	0.0002	0.0033	0.0013	0.00045	0.0029	0.0015
**IQR3**	0.0130	0.0153	0.0121	0.0123	0.0104	0.0044
**TAURINE KONJUGATES**						
**MEDIAN**	36.46	26.35	15.10	33.13	20.77	14.39
**IQR1**	30.71	15.44	14.52	27.51	15.62	10.81
**IQR3**	48.57	38.07	34.92	38.83	22.13	18.21
**GLYCINE KONJUGATES**						
**MEDIAN**	63.18	73.42	84.41	66.59	78.91	85.48
**IQR1**	51.33	61.85	65.01	61.09	77.74	81.63
**IQR3**	69.15	84.26	85.26	72.31	84.19	89.00

Abbreviations: CA *Cholic acid*; CDCA *Chenodeoxycholic acid*; DCA *Deoxycholic acid*; DHCA *Dehydrocholic acid*; GHCA *Glycohyocholic acid*; HOPE *Hypothermic oxygenated machine perfusion*; LCA *Lithocholic acid*; POD *Postoperative day*; SCS *Static cold storage*; TMCA *Tauro-muricholic acids*; UCA *Ursocholic acid*; UDCA *Ursodeoxycholic acid*

Table S5 Intensities of biliary phosphatidylcholine (PC) and phosphatidylethanolamine (PE), and the biliary BA/PC ratio

	SCS			HOPE		
**POD**	1	2	3	1	2	3
**PC**						
**MEDIAN**	4.06E+08	6.29E+08	4.02E+08	4.39E+08	6.26E+08	6.49E+08
**IQR1**	1.98E+08	4.77E+08	2.34E+08	3.75E+08	3.91E+08	5.53E+08
**IQR3**	7.08E+08	7.76E+08	6.05E+08	5.32E+08	8.52E+08	9.31E+08
**PE**						
**MEDIAN**	4.04E+08	5.89E+08	3.93E+08	4.19E+08	5.38E+08	5.25E+08
**IQR1**	2.20E+08	3.84E+08	2.63E+08	3.14E+08	3.34E+08	5.09E+08
**IQR3**	4.81E+08	7.05E+08	5.70E+08	5.10E+08	6.15E+08	7.27E+08
**BA/PC**						
**MEDIAN**	6.98	3.99	4.62	5.68	3.34	3.20
**IQR1**	3.16	2.89	3.28	4.70	2.86	2.69
**IQR3**	8.85	5.71	7.52	6.30	7.23	4.47

Abbreviations: BA, bile acid; PC, Phosphatidylcholine; PE, Phosphatidylethanolamine; PL, Phospholipids; HOPE, Hypothermic oxygenated machine perfusion; POD, Postoperative day; SCS, Static cold storage

Table S6: Univariable logistic and Cox regression on biliary bile acids and clinical outcomes

Univariate Regression							
	AUC[#]	Acute cellular rejection°		Biliary complications°		1-year Graft Survival	
**Total bile acids**	$4.38 \times 10^9 \pm 7.91 \times 10^8$	1.619 (0.689-3.804)	p 0.26	1.161 (0.442 – 3.051)	p 0.76	0.818 (0.287 – 2.328)	p 0.70
**Hydrophobic bile acids**	$1.76 \times 10^9 \pm 6.73 \times 10^8$	1.222 (0.524-2.849)	p 0.64	1.760 (0.640 –4.845)	p 0.27	1.949 (0.759 – 5.002)	p 0.16
**Hydrophilic bile acids**	$7.68 \times 10^8 \pm 3.32 \times 10^8$	0.651 (0.253 – 1.671)	p 0.37	0..593 (0.189 – 1.863)	p 0.37	0.615 (0.190 – 1.997)	p 0.41

Multivariate Regression[2]						
	Acute cellular rejection°		Biliary complications°		1-year Graft Survival	
**Total bile acids**	1.872 (0.757 – 4.630)	p 0.17	1.418 (0.499 – 4.034)	p 0.51	1.111 (0.390 – 3.163)	p 0.84
**Hydrophobic bile acids**	1.264 (0.527 – 1.264)	p 0.60	2.051 (0.547 – 7.687)	p 0.28	0.492 (0.099 – 2.443)	p 0.23
**Hydrophilic bile acids**	0.731 (0.253 – 2.111)	p 0.56	0.649 (0.149 – 2.829)	p 0.56	1.227 (0.384 – 3.914)	p 0.73

#Area under the Curve of cumulative bile acid intensities calculated through trapezoidal method; Values are reported as Median ± Standard deviation

*Regressions Model were performed based on the z-Score of Bile acid intensities, to allow for a more precise model. Values are reported as Odds or Hazard ratio (95% Confidence Interval).

° at 90 days after liver transplantation

[2]Adjusted for donor age, cold and warm ischemia time, macrosteatosis, and intraoperative red blood cell transfusions

Table S7 Recipient and donor baseline characteristics of patients included in the matched serum bile acids analysis

Characteristics	CCS (n = 8)	HOPE (n = 8)	*p*
Donor Characteristics			
Age, y, median (IQR)	69.0 ± 15.25	69.0 ± 17.05	0.959
BMI, kg/m^2, median (IQR)	28.5 ± 2.80	28.5 ± 5.67	>0.999
Sex, female: male, n (%)	4 (50%): 4 (50%)	4 (50%): 4 (50%)	>0.999
ET-DRI Score*	2.07 ± 0.45	1.91 ± 0.36	0.721
Cause of death n (%)			0.243
Cerebrovascular accident	4 (50%)	5 (62.5%)	
Anoxia	1 (12.5%)	3 (37.5%)	
Trauma	3 (37.5%)	0	
ECD-Criteria			
Donor age ≥65	4 (50%)	6 (75%)	0.608
Donor ICU ≥7 days	0	3 (37%)	0.200
Donor BMI >30 kg/m^2	2 (25%)	2 (25%)	>0.999
Macro/mixed steatosis >40%	2 (25%)	2 (25%)	>0.999
Serum sodium >165 mmol/L	1 (4%)	2 (10%)	>0.572
Serum AST/ALT >3× of normal	3 (37,5%)	2 (25%)	>0.999
Serum bilirubin >2 mg/dL	0	1 (12.5%)	>0.999
Recipient Characteristics			
Age, y, median (IQR)	60.5 ± 5.28	59.0 ± 5.344	0.878
BMI, kg/m^2, median (IQR)	27.5 ± 2.64	28.5 ± 4.05	0.505
Sex, female: male, n (%)	2 (25%): 6 (75%)	2 (25%): 6 (75%)	>0.999
LabMELD, median (IQR)	19.0 ± 10.01	13.5 ± 9.93	0.328
BAR Score, median (IQR)§	8.5 ± 4.91	5.5 ± 5.34	0.721
Listing indications, n (%)			0.490
HCC in cryptogenic cirrhosis	4 (50%)	1 (12.5%)	
HCC in NASH cirrhosis	3 (37.5%)	5 (62.5%)	
Other	1 (12.5%)	2 (25%)	
Preservation details			
Total cold preservation time, min, median (IQR)	560.5 ± 88.55	487.5 ± 85.10	0.442
Warm ischemic, min, median (IQR)	51.0 ± 9.48	43.5 ± 8.25	0.279

Data presented as median and interquartile range if not noted otherwise.
Mann-Whitney U Test was used for continuous variables, Chi Square Test or two-sided Fisher's exact test for categorical variables.
Abbreviations: ALT alanine aminotransferase, AST aspartate aminotransferase, BAR Balance of Risk Score, BMI body mass index, ECD extended criteria donor, ET-DRI Eurotransplant Donor Risk Index, HCC hepatocellular carcinoma, HOPE Hypothermic, oxygenated machine perfusion; ICU intensive care unit, IQR interquartile range, LabMELD laboratory Model for End-Stage Liver Disease, MASH metabolic dysfunction–associated steatohepatitis; PSC primary sclerosing cholangitis, PBC, primary biliary cholangitis, SCS Static cold storage

Table S8 Hydrophobicity Index* (IQR) of biliary bile acid composition

POD	***SCS***	***HOPE***	***p***
1	0.22 (0.149-0.285)	0.19 (0.149-0.281)	0.389
2	0.11 (0.07-0.26)	0.11 (0.08-0.19)	0.587
3	0.10 (0.05-0.23)	0.14 (0.10-0.18)	0.899

* calculated according to Heumann et al.[40] based on PA-fraction

Table S9 Patient and Donor baseline characteristics between HOPE-ECD-DBD participants receiving or not receiving T-Drainage intraoperatively

Characteristics	Inserted T-Drainage (n = 26)	No T-Drainage (n = 20)	*p*
Donor Characteristics			
Age, y, median (IQR)	69 (59 – 80)	73 (61 – 78)	0.387
BMI, kg/m^2, median (IQR)	28 (24 – 31)	28 (25 – 32)	0.973
Sex, female: male, n (%)	12 (46.2%): 14 (53.8%)	9 (45%): 11 (55%)	0.588
ET-DRI Score*	2.050 (1.82 – 2.16)	2.055 (1.92 – 2.33)	0.369
Cause of death n (%)			
Cerebrovascular accident	14 (54%)	16 (80%)	0.117
Anoxia	8 (31%)	2 (10%)	0.150
Trauma	4 (15%)	2 (10 %)	0.684
Donor Resuscitation, yes, n (%)	9 (35%)	5 (25%)	0.535
ECD-Criteria			
Donor age ≥65	16 (62%)	15 (75%)	0.365
Donor ICU ≥7 days	6 (23%)	7 (35%)	0.511
Donor BMI >30 kg/m^2	7 (27%)	6 (30%)	>0.999
Macro/mixed steatosis >40%	6 (23%)	4 (20%)	>0.999
Serum sodium >165 mmol/L	1 (4%)	2 (10%)	>0.572
Serum AST/ALT >3× of normal	9 (35%)	5 (25%)	0.535
Serum bilirubin >2 mg/dL	2 (8%)	1 (5%)	>0.999
No. of fulfilled ECD-Criteria			
1 Of 7	9 (35%)	4 (20%)	0.336
2–3 Of 7	14 (54%)	12 (60%)	0.769
>3 Of 7	3 (12%)	4 (20%)	0.682
Macrosteatosis, n (%)			
None to mild (0–29%)	23 (92%)	16 (94%)	>0.999
Moderate to severe (≥30%)	2 (8%)	1 (6%)	>0.999
Recipient Characteristics			
Age, y, median (IQR)	62 (54–65)	61 (56–66)	0.982
BMI, kg/m^2, median (IQR)	28 (26–29)	28 (24–34)	0.622
Sex, female: male, n (%)	4 (15%): 22 (85%)	4 (20%): 16 (80%)	0.489
LabMELD, median (IQR)	14 (8–25)	16 (9–24)	0.610
BAR Score, median (IQR)§	6 (4–12)	5 (2–10)	0.333
Listing indications, n (%)			
Alcoholic cirrhosis	3 (12%)	8 (40%)	0.038
Viral cirrhosis	0 (0%)	3 (15%)	0.075
HCC in cirrhosis	16 (62%)	4 (20%)	0.007
PSC/PBC	2 (8%)	1 (5%)	>0.686
Other	6 (23%)	4 (20%)	0.547

Data presented as median and interquartile range if not noted otherwise.
Mann-Whitney U Test was used for continuous variables, Chi Square Test or two-sided Fisher's exact test for categorical variables.
Abbreviations: ALT alanine aminotransferase, AST aspartate aminotransferase, BAR Balance of Risk Score, BMI body mass index, ECD extended criteria donor, ET-DRI Eurotransplant Donor Risk Index, HCC hepatocellular carcinoma, HOPE Hypothermic, oxygenated machine perfusion; ICU intensive care unit, IQR interquartile range, LabMELD laboratory Model for End-Stage Liver Disease, MASH metabolic dysfunction–associated steatohepatitis; PSC primary sclerosing cholangitis, PBC, primary biliary cholangitis, SCS Static cold storage

Table S10: Intrahepatic bile acids levels before (T1) and after reperfusion (T2)

	Timepoint	Group	Median*	Standard Deviation*
TBA	T1	SCS	8.7101	5.96455
TBA	T1	HOPE	8.3310	4.53164
TBA	T2	SCS	10.7725	4.87970
TBA	T2	HOPE	10.3069	4.92967
HOBA	T1	SCS	5.9697	4.94508
HOBA	T1	HOPE	5.8148	3.24662
HOBA	T2	SCS	6.9636	3.98412
HOBA	T2	HOPE	6.0833	3.65536
PBA	T1	SCS	4.9334	2.90423
PBA	T1	HOPE	5.6612	2.94105
PBA	T2	SCS	6.6650	2.41317
PBA	T2	HOPE	6.0662	4.09755
SBA	T1	SCS	3.1947	3.61722
SBA	T1	HOPE	2.7832	2.33999
SBA	T2	SCS	3.4233	3.18572
SBA	T2	HOPE	2.9525	2.72602
CA	T1	SCS	2.8428	1.46329
CA	T1	HOPE	3.1368	1.71486
CA	T2	SCS	3.2362	1.77026
CA	T2	HOPE	3.6828	2.39526
CDCA	T1	SCS	2.0906	1.61213
CDCA	T1	HOPE	2.6089	1.33094
CDCA	T2	SCS	3.2584	1.10055
CDCA	T2	HOPE	2.9068	2.03723
DCA	T1	SCS	3.1379	0.87679
DCA	T1	HOPE	2.72	0.56645
DCA	T2	SCS	3.3024	0.77131
DCA	T2	HOPE	2.8886	0.65837
LCA	T1	SCS	0.0466	0.01556
LCA	T1	HOPE	0.0466	0.00894
LCA	T2	SCS	0.0403	0.00632
LCA	T2	HOPE	0.0377	0.00807
UDCA	T1	SCS	0.0177	0.00207
UDCA	T1	HOPE	0.018	0.00112
UDCA	T2	SCS	0.019	0.00468
UDCA	T2	HOPE	0.02	0.00801

* Results are shown as pmol/mg

Abbreviations: CA Cholic acid; CDCA Chenodeoxycholic acid; DCA Deoxycholic acid; HOBA Hydrophobic bile acids; HOPE Hypothermic oxygenated machine perfusion; LCA Lithocholic acid; POD Postoperative day; PBA Primary bile acids; SCS Static cold storage; SBA Secondary bile acids; TBA Total bile acids UDCA Ursodeoxycholic acid

Table S11 Serum bile acid levels in µmol/l of the analyzed subcohort (n=16)

Timepoint	Group (n=8 per Group)	TBAs (Median ± SD)	PBA (Median ± SD)	SBA (Median ± SD)	HOBA (Median ± SD)
Admission	SCS	190.46 ± 126.82	128.33 ± 97.84	12.88 ± 91.31	115.34 ± 67.52
Admission	HOPE	165.96 ± 143.95	146.06 ± 152.06	19.22 ± 20.47	93.99 ± 61.13
POD-1	SCS	57.25 ± 257.88	24.69 ± 227.92	22.76 ± 41.14	18.93 ± 66.95
POD-1	HOPE	30.61 ± 14.81	14.79 ± 11.08	5.72 ± 14.08	6.81 ± 4.10
POD-2	SCS	40.82 ± 58.50	30.66 ± 55.86	13.59 ± 6.85	10.50 ± 19.72
POD-2	HOPE	21.68 ± 38.42	16.66 ± 11.64	5.45 ± 28.03	4.52 ± 3.78
POD-3	SCS	31.60 ± 26.68	22.31 ± 25.23	7.95 ± 4.87	9.20 ± 7.25
POD-3	HOPE	30.91 ± 13.03	27.05 ± 11.08	4.3 ± 6.55	7.19 ± 13.17

Abbreviations: HOPE Hypothermic oxygenated machine perfusion; HOBA Hydrophobic bile acids: POD Postoperative day; PBA Primary bile acids; SCS Static cold storage; SBA Secondary bile acids; TBA Total bile acids
