# Supplementary material for: Effects of hypothermic oxygenated machine perfusion on bile composition after liver transplantation – Findings from a randomized controlled trial
Source: JHEP Rep. 2025 Oct 17;8(2):101647. doi: 10.1016/j.jhepr.2025.101647 (PMC12810548; doi:10.1016/j.jhepr.2025.101647)
Supplement: Multimedia component 2 [file mmc2.docx]

**JHEP Reports**

**CTAT methods**

Tables for a “Complete, Transparent, Accurate and Timely account” (CTAT) are now mandatory for all revised submissions. The aim is to enhance the reproducibility of methods.

- Only include the parts relevant to your study
- Refer to the CTAT in the main text as ‘Supplementary CTAT Table’
- Do not add subheadings
- Add as many rows as needed to include all information
- Only include one item per row

**If the CTAT form is not relevant to your study, please outline the reasons why:**

|  |
| --- |

- 1. **Antibodies**

| **Name** | **Citation** | **Supplier** | **Cat no.** | **Clone no.** |
| --- | --- | --- | --- | --- |
| Not applicable |  |  |  |  |

- 1. **Cell lines**

| **Name** | **Citation** | **Supplier** | **Cat no.** | **Passage no.** | **Authentication test method** |
| --- | --- | --- | --- | --- | --- |
| Not applicable |  |  |  |  |  |

- 1. **Organisms**

| **Name** | **Citation** | **Supplier** | **Strain** | **Sex** | **Age** | **Overall n number** |
| --- | --- | --- | --- | --- | --- | --- |
| Not applicable |  |  |  |  |  |  |

- 1. **Sequence based reagents**

| **Name** | **Sequence** | **Supplier** |
| --- | --- | --- |
| Custom SYBR Green–based qPCR assays | Please refer to Supplementary Table 2 | SYBR Green detection |

- 1. **Biological samples**

| **Description** | **Source** | **Identifier** |
| --- | --- | --- |
| Liver tissue, blood and bile samples acquired during the HOPE-ECD-DBD trial | Department of Surgery, Charité Universitätsmedizin Berlin, Germany  Department of Surgery and Transplantation, University Hospital RWTH Aachen, Germany  Department of General, Visceral, and Transplant Surgery, Ludwig-Maximilians-University Munich, Germany  Department of Transplantation Surgery, Institute for Clinical and Experimental Medicine, Prague, Czech Republic | Not applicable |

- 1. **Deposited data**

| **Name of repository** | **Identifier** | **Link** |
| --- | --- | --- |
| PeptideAtlas (metabolomics data optained from LC-MS/MS analysis) | PASS05891 | http://www.peptideatlas.org/PASS/PASS05891 |

- 1. **Software**

| **Software name** | **Manufacturer** | **Version** |
| --- | --- | --- |
| MultiQuant™ Software | Sciex | v.2.1.1 |
| GraphPad Prism | Dotmatics | v.10.2.3 |
| SPSS Statistics | IBM Corp., Armonk, NY | v.29 |

- 1. **Other (*e.g*. drugs, proteins, vectors etc.)**

| Not applicable |  |  |
| --- | --- | --- |
|  |  |  |

- 1. **Please provide the details of the corresponding methods author for the manuscript:**

| Frederik Schliephake  Im Neuenheimer Feld  420 Heidelberg  Universitätsklinik Heidelberg  frederik.schliephake@med.uni-heidelberg.de  +4930450553179 |
| --- |

**2.0 Please confirm for randomised controlled trials all versions of the clinical protocol are included in the submission. These will be published online as supplementary information.**

| Not applicable |
| --- |
